# The impact of environmental change on Palaeolithic and Mesolithic plant use and the transition to agriculture at Franchthi Cave, Greece

**DOI:** 10.1371/journal.pone.0207805

**Published:** 2018-11-20

**Authors:** Eleni Asouti, Maria Ntinou, Ceren Kabukcu

**Affiliations:** 1 Department of Archaeology, Classics and Egyptology, University of Liverpool, Liverpool, United Kingdom; 2 M.H. Wiener Laboratory for Archaeological Science, American School of Classical Studies at Athens, Athens, Greece; Max Planck Institute for the Science of Human History, GERMANY

## Abstract

The multi-period (~38,000–6000 cal BP) site of Franchthi Cave, located in the Argolid peninsula of southern mainland Greece, is unique in the Eastern Mediterranean for preserving a long archaeological sequence extending from the Upper Palaeolithic through to the end of the Neolithic period. In this paper, we present new anthracological (carbonized fuel wood waste) evidence from Franchthi Cave with which we reconstruct the changing ecology of woodland vegetation in its environs during the late Pleistocene and the early-mid Holocene. The integrated archaeobotanical record (charred wood and non-wood macro-remains) demonstrates that in the Lateglacial the now-submerged coastal shelf of the southern Argolid peninsula was covered by steppe grassland vegetation dominated by junipers, almonds, cereals and legumes. The rapid climatic amelioration that marked the start of the Holocene brought about the disappearance of juniper and the expansion of deciduous woodland, cereals and lentils. This woodland-grassland biome bears no analogues in the modern and historical vegetation ecology of the Aegean basin. Instead, it is directly comparable to the steppe woodland biomes exploited by late Pleistocene and early Holocene hunter-gatherers in Southwest Asia, and points to the convergent evolution of late Pleistocene and early Holocene plant exploitation strategies between the two regions. Continuous sea-level rise during the early Holocene led to the gradual extinction of this unique palaeohabitat, which acted as the catalyst for the selective introduction of domesticated cereal crops at Franchthi Cave in the early 9^th^ millennium cal BP. Our meta-analysis of the non-wood archaeobotanical data puts into question the concept of the wholesale introduction of a crop “package” by pioneer settler groups arriving from the East. It is proposed instead that selective cereal crop introduction formed part of a complex pattern of sociocultural interactions that brought together indigenous and immigrant groups into new communities.

## Introduction

Across the Mediterranean basin the period between the end of the last Ice Age at ~20 ka BP and the early Holocene (~11.7–8.2 ka BP) was marked by dramatic environmental transformations caused by the twin impacts of postglacial climatic amelioration and sea-level rise (SLR). Globally, this period was characterized by high SLR rates (~12–15 mm/yr) which increased significantly (≥40 mm/year) during Meltwater Pulse-1A (MWP-1A) spanning the first 500 years of the Greenland Interstadial-1 (GI-1 ~14.5–12.9 ka BP) [1]. The impact of these global events was particularly pronounced in the southern Aegean where current models of postglacial relative SLR project a vast amount of land inundation to have occurred between ~15–9 ka BP [2]. However, little is still known about how climate change and SLR affected terrestrial biomes in the southern Aegean during the Lateglacial and the early Holocene. A major issue for reconstructing the evolution of late Palaeolithic and Mesolithic lifeways during this critical period is the extent to which the inundation of the coastal shelf zone affected the biotic resources exploited by hunter-gatherers for food and fuel. Terrestrial and near-shore pollen records dating to the Lateglacial and the start of the Holocene are virtually absent from the southern Aegean due to poor organic preservation and the low chronological resolution of the few available sequences [3]. Furthermore, marine offshore pollen sequences cannot capture local-scale terrestrial vegetation dynamics due to their very large catchment areas (see, for example, the study published by Triantaphyllou *et al*. [4]). There are thus no palaeobotanical data from the southern Aegean with which to reconstruct the evolution of terrestrial plant ecologies and people-plant interactions associated with these major environmental transformations.

**Fig 1:**
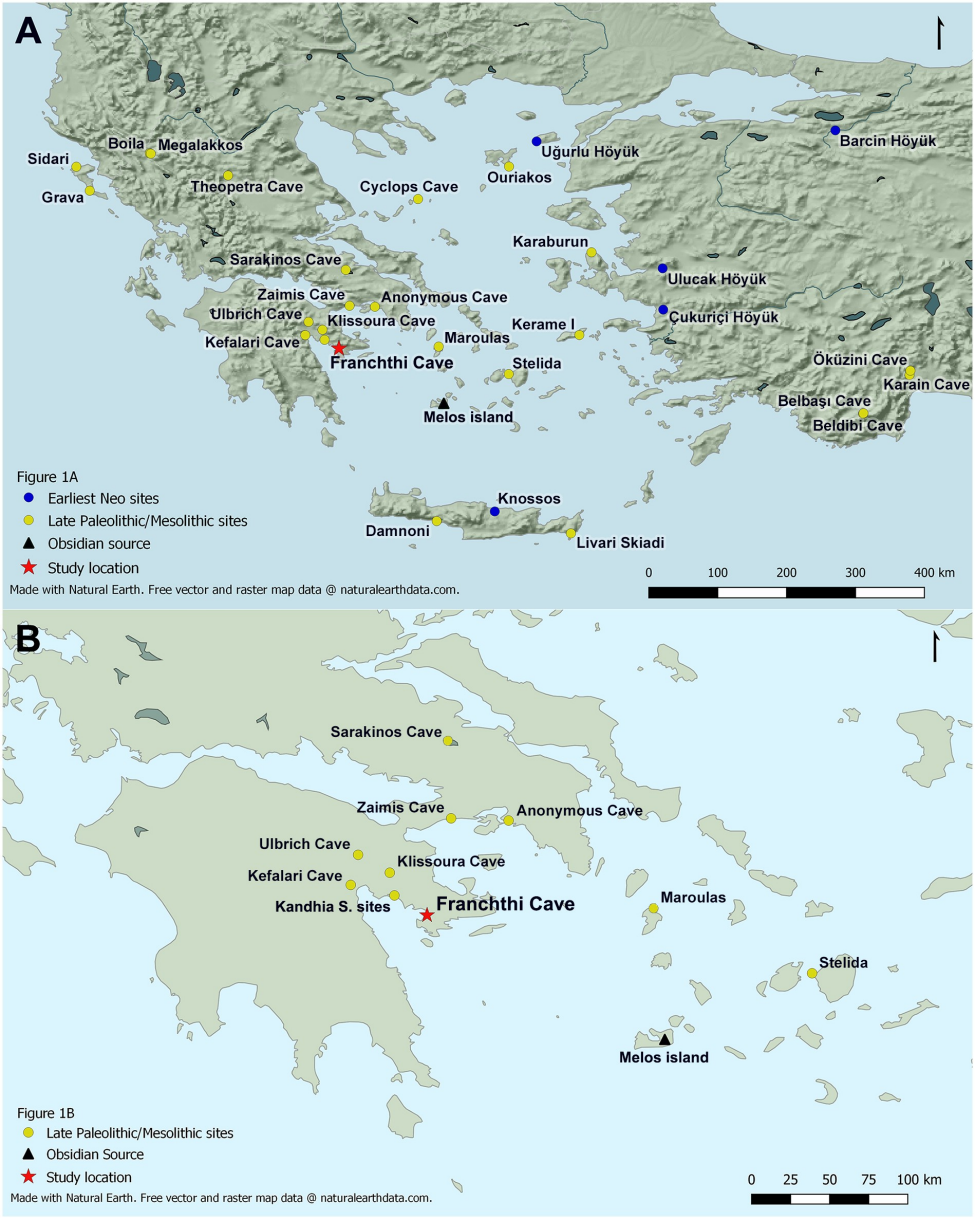
Maps showing locations of Upper Palaeolithic, Mesolithic and earliest Neolithic sites in the Aegean basin. (A) Map of Late Palaeolithic, Mesolithic and earliest Neolithic sites in the Aegean basin. (B) Detail of distribution of Late Palaeolithic and Mesolithic sites in the Argolid peninsula and neighboring areas (Map made using QGIS version 3.2; Source: Natural Earth, Physical map layers and raster layers from 1:10).

**Fig 2:**
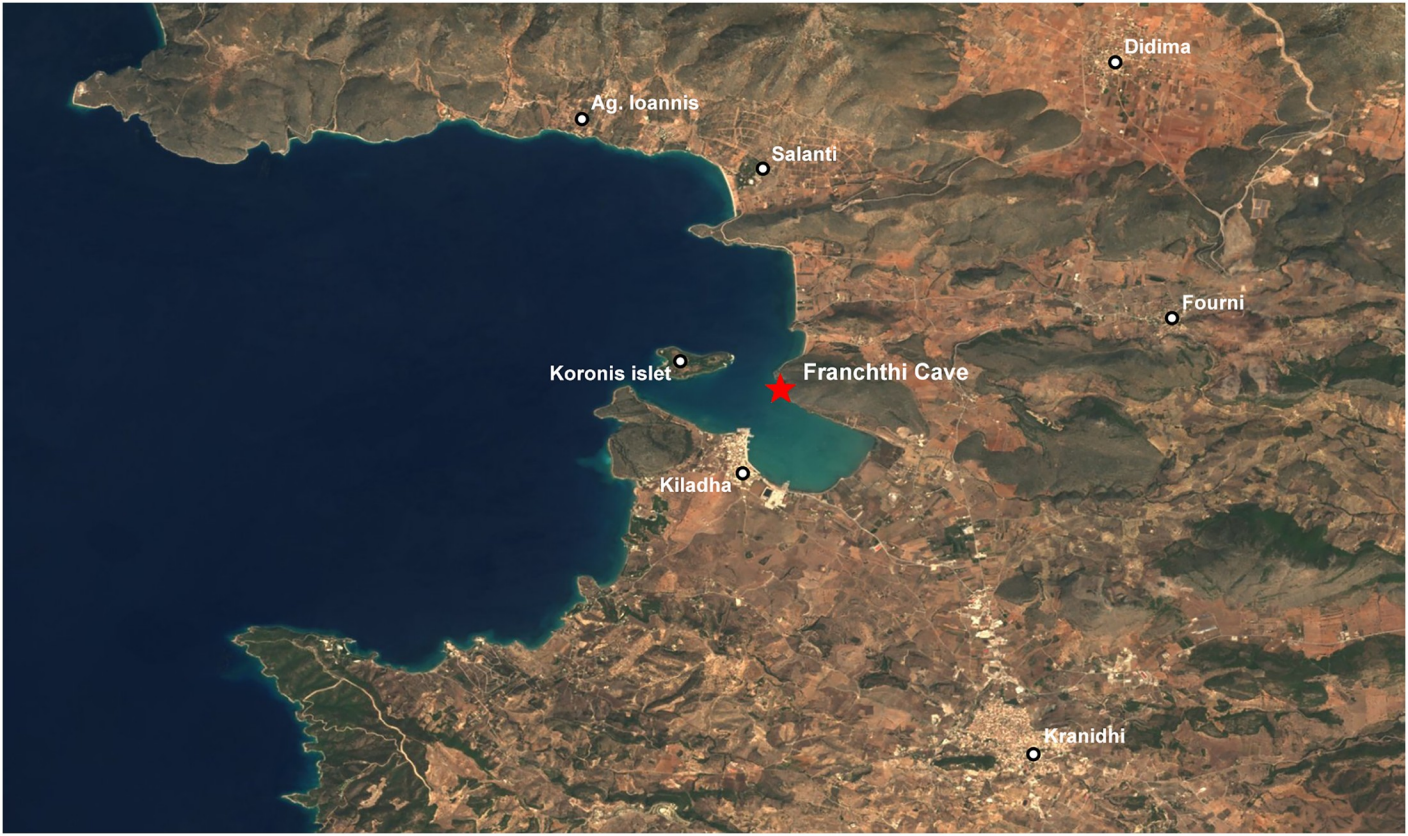
Location of FC on the Kiladha Bay of the southern Argolid peninsula. Sentinel-2 cloudless Satellite imagery database, under CC BY 4.0—https://s2maps.eu by EOX IT Services GmbH (Contains modified Copernicus Sentinel data 2016 & 2017).

Although recurrent field surveys in the Aegean have increased significantly the number of late Palaeolithic and Mesolithic sites known from this area (see Fig 1A and 1B) the number of excavations remains comparatively low (see recent overviews by Sampson [5] and Çilingiroğlu et al. [6]). Additionally, in the southern Aegean most late Palaeolithic and Mesolithic sites sampled for archaeobotanical remains have produced scanty non-wood but more abundant wood charcoal macro-remains [7–9]. In general, poor archaeobotanical preservation is accentuated in coastal open-air sites and shallow caves/rock-shelters, due to their sedimentary environments (characterized by marked seasonal fluctuations in soil moisture) which severely hinder the preservation of fragile charred plant remains [10]. A notable exception to this situation is Franchthi Cave (FC). Located on the northeastern coast of the Peloponnese (Figs 1B and 2) the site was excavated between 1967–1976 by an international team led by T.W. Jacobsen of Indiana University. FC is unique in the Eastern Mediterranean for preserving a long archaeological sequence extending from the Upper Palaeolithic through to the Mesolithic and the Neolithic periods (~38,000–6000 cal BP) [11–12]. Its stratigraphy spans two key periods that witnessed major socioeconomic transformations in the history of humanity: the population expansion and intensification of economic activities that took place after the end of the Last Glacial Maximum, and the transition from foraging to farming that characterized the first half of the Holocene. FC provides several distinctive advantages for palaeovegetation reconstruction including adequate preservation and sampling of archaeobotanical remains, alongside a reasonably robust radiometric chronology that permits correlating its archaeological sequence to regional and global SLR models and palaeoclimatic archives [13–16]. FC thus affords a research framework that, to date, remains unique in the southern Aegean with which to reconstruct the evolution of the regional late Pleistocene and early Holocene vegetation ecologies and prehistoric plant use.

Previous studies of non-wood (seeds, fruits and nuts) charred plant remains from FC suggested that successive episodes of climate change from the Lateglacial through to the early Holocene exerted minimal impacts on the availability, distribution and use of plant-food resources [14, 17]. Through the late Upper/Final Palaeolithic and Mesolithic periods, little variation could be discerned in the species composition of the archaeobotanical samples that was interpreted to reflect the postglacial gradual spread of Mediterranean woodland vegetation on the southern Argolid coastal lowlands, much akin to the present-day vegetation in this area [14]. During the same period, the study of faunal remains indicated the increasing importance of snails and marine resources that was linked to environmental pressures on terrestrial game associated with SLR [18]. Somewhat paradoxically, SLR was not considered to have exerted appreciable impacts on the distribution of plant-food resources. Furthermore, the introduction of domesticated cereal crops and animals in the early 9th millennium cal BP has been widely interpreted as a manifestation of the displacement of local Mesolithic forager-fisher-hunter lifeways by an *ex oriente* arrived mature agricultural economy [14, 19]. However, radiocarbon dates recently obtained on crop seeds have also pinpointed the possibility that domesticated cereal crops were adopted in the first instance by indigenous Mesolithic groups [20]. In this framework, it remains unknown whether crop introduction could have been motivated by the degradation and gradual extinction of traditionally used wild plant resources resulting from the inundation of the southern Argolid coastal shelf. The FC archaeobotanical record has provided tantalizing clues for the potential existence locally of Lateglacial and early Holocene palaeohabitats for which there are no analogues in the late Holocene regional vegetation history. This is suggested, for example, by the predominant presence in late Upper/Final Palaeolithic and Mesolithic botanical remains of *Amygdalus* (almond) and *Pistacia* (terebinth) nuts alongside wild-type cereals (*Avena*/oats, *Hordeum*/barley) and pulses (*Lens*/lentils). In effect, the closest archaeobotanical parallels to the FC pre-Neolithic plant-food spectrum lay not with the Mediterranean ecoregions of mainland Greece, the Aegean islands, and the western and southern Anatolian coasts but, rather strikingly, with the late Pleistocene and early Holocene botanical spectra originating in the steppe and open woodland-grassland biomes of continental Southwest Asia [21–22]. However, the potential held by high-resolution archaeobotanical data for revisiting the diversity of the southern Aegean postglacial plant ecologies, and how the latter influenced local and regional pathways from foraging to farming, has remained largely unexplored.

The principal factor constraining the utility of the FC non-wood botanical remains for palaeoenvironmental reconstruction is that they represent primarily the debris of prehistoric plant-food use, which is determined (as is the case with all human food consumption) by a multitude of factors other than species availability in the site environs. Given the lack of well-preserved and radiocarbon dated pollen sequences, the sole viable method for reconstructing palaeovegetation at temporal scales approximating those of prehistoric habitation is through charcoal science (anthracology): the study of wood charcoal macro-remains originating from routine fuel wood use, which are retrieved from stratified archaeological deposits. Unlike plant-food remains, charred fuel wood waste can be used to generate high-resolution reconstructions of the floristic composition and palaeoecology of ancient woodlands, subject to the application of appropriate analysis and taphonomic evaluation protocols [23]. This paper presents the results of the first application of anthracology to FC, on fuel wood macro-remains covering the timespan ~28,000–6000 cal BP. To enhance further the precision of the resulting palaeovegetation reconstructions, we integrated the new wood charcoal data with previously published non-wood (seed/fruit/nut) charred remains originating from the same botanical samples. In addition, we performed a multivariate analysis of the FC non-wood archaeobotanical data corpus originally published by Julie Hansen [14] (including all botanical samples regardless of the availability of wood charcoal data). These integrative analyses permitted exploring in unprecedented detail the regional postglacial palaeohabitats in which arboreal and herbaceous floras co-existed, how and when they became established in the site hinterland, and how they were shaped by climate change and SLR during the long history of human habitation at FC. Crucially, they have also provided new insights into the ecological context of the first introduction of crop domesticates in the southern Greek mainland and illuminated further some of the more idiosyncratic aspects of FC Neolithic plant use.

## Results

### Excavations at FC: Stratigraphy, chronology and phasing

**Fig 3:**
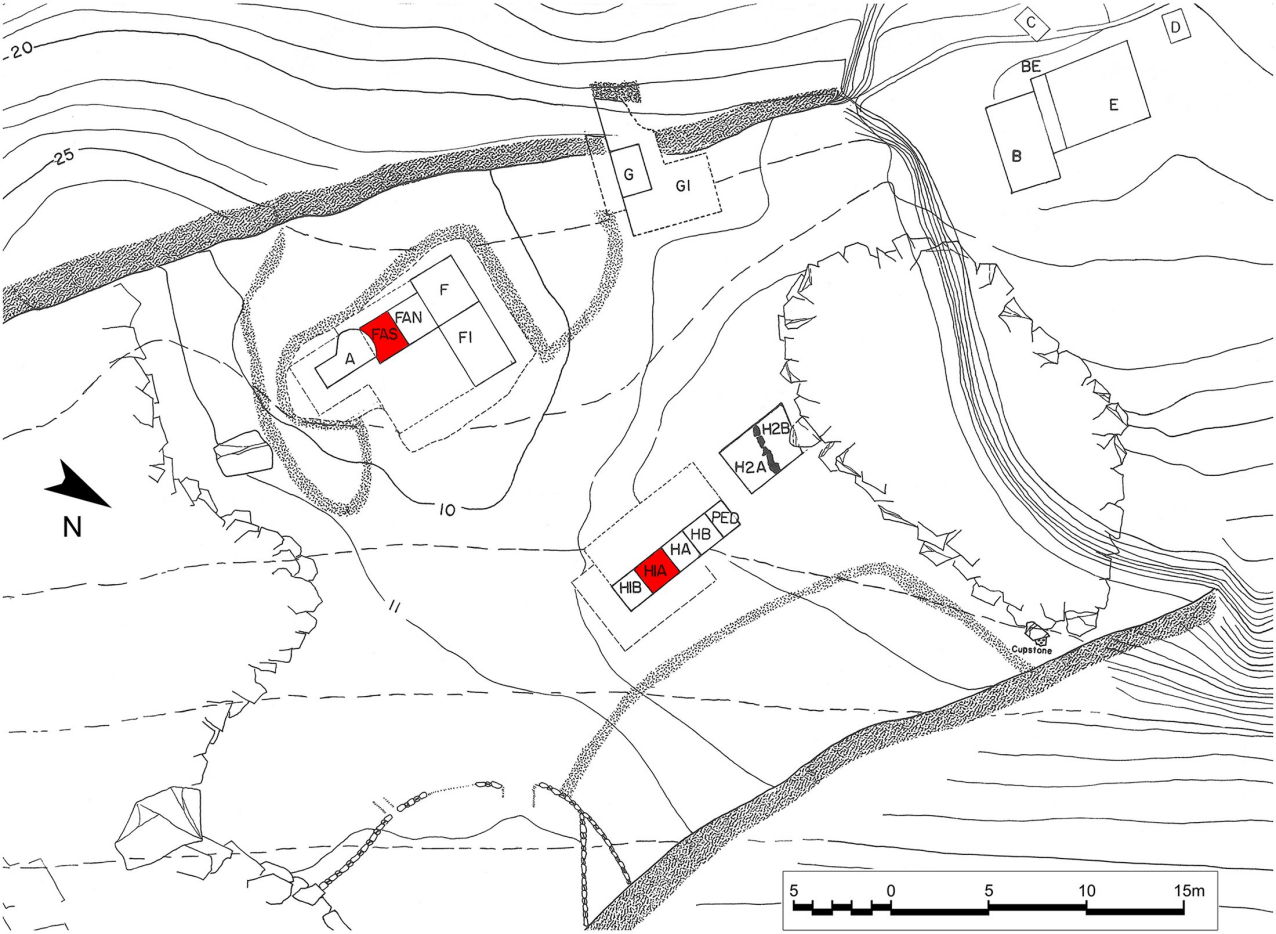
Plan of excavated areas at Franchthi Cave. Sampled trenches are marked in red.

FC forms a long and partly collapsed chamber situated at the northwestern end of a rocky limestone promontory, which is engulfed by low-lying alluvium and slopewash colluvial deposits (Fig 2). It is flanked on its seaward side by numerous karstic freshwater springs that are presently submerged below sea level but were probably accessible during the prehistoric periods. Excavations inside the cave chamber were conducted in four trenches (A, F, G, H covering ~136.5m^2^ or 10% of the total accessible area) (Fig 3). They uncovered a long archaeological sequence including Upper Palaeolithic (Aurignacian, “Mediterranean Gravettian”), Late and Final Palaeolithic (“Epigravettian”), Mesolithic (Lower, Upper, Final) and Neolithic phases. The maximum depth of the excavated deposits containing cultural materials was ~11m without reaching the bedrock [11]. A Campanian tephra layer found in the deepest excavated layers in trenches FA and HH1 has been dated by 40Ar/39Ar to ~39,000 years ago. Thus, it is highly likely that early prehistoric habitation at FC may extend as far back as the Middle Palaeolithic.

The FC archaeological sequence is subdivided in several lithostratigraphic units (strata) that provide the principle means for stratigraphic correlation between the different trenches [11, 20]. Archaeological phasing (Franchthi General Phases/FGPs) for the FC pre-Neolithic habitations is provided by a synthetic framework developed by Catherine Perlès based on the data available on sediments, mammals, fish, land snails, marine mollusks, non-wood botanical remains, ornaments and lithics, and (for the Neolithic habitations) on lithic and ceramic techno-typological classifications (the latter are denoted as Cultural Phases/CPs) [24–25]. The earliest part of the archaeological sequence from which wood charcoal remains were available for analysis is radiocarbon dated at ~28,000 cal BP (stratum R/FGP II followed by the undated stratum S1/FGP III, both characterized by sporadic occupation). There was a long hiatus between ~24/23,000 and 15,000 cal BP that marked the onset of S2/FGP IV. 3 major occupation phases are distinguished after ~15,000 cal BP, punctuated by brief hiatuses: the late Upper/Final Palaeolithic of strata T (T1-3/FGP V) and U-V (FGP VI), the Mesolithic of strata W (W1-3/FGP VII) and X (X1-2/FGP VIII), and the Neolithic of stratum Y (Y1-3/CPs N.I-V). The upper end of the sequence is radiocarbon dated to the end of the Neolithic period (~6000 cal BP) [11, 13–14, 24–25].

The analysis of animal bone taphonomy and diachronic trends in prey diversity has concluded that a pattern of hunting both small- and large-bodied mammals (characterized by different capture costs and environmental preferences) pervades the FC pre-Neolithic sequence [18]. For this reason, even though occupation intensity varied widely through time, it is likely that the bulk of the FC pre-Neolithic habitations represent residential camps containing the remains of a palimpsest of daily activities (including routine fuel wood collection and use) rather than temporarily occupied specialist-task sites (which would have been associated with episodic fire use). The results of faunal analyses thus lend additional support to the ecological representativeness of the FC anthracological remains as reliable proxies for reconstructing local woodland composition and ecology, and their changes through time.

### The charcoal stratigraphy

**Fig 4:**
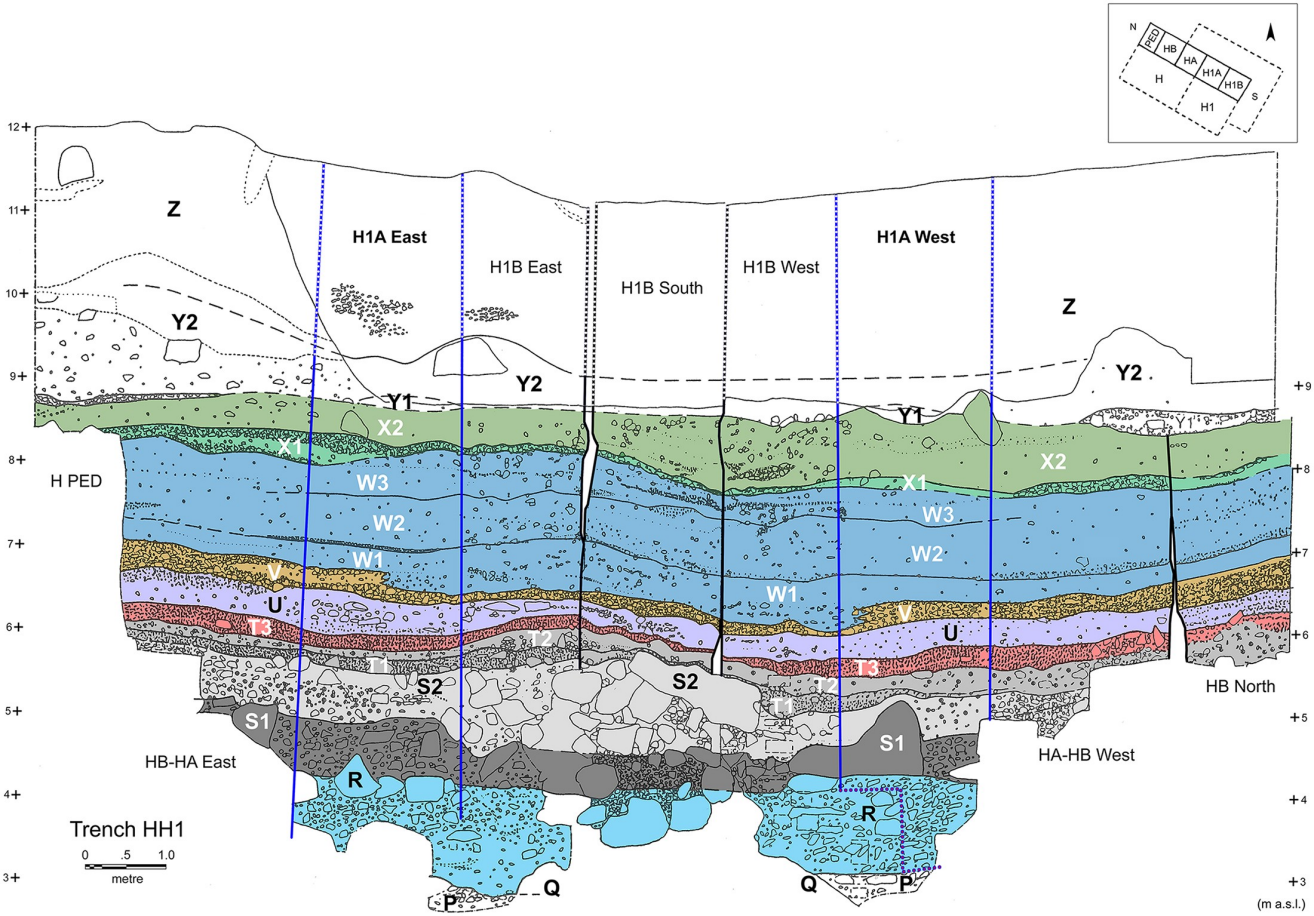
Trench HH1 composite section highlighting key FC strata represented in the anthracological assemblage. Trench H1A sections are demarcated with blue lines (redrawn based on data presented in Farrand 2000 [11]: Plates 13–14 and Fig 4.2).

**Fig 5:**
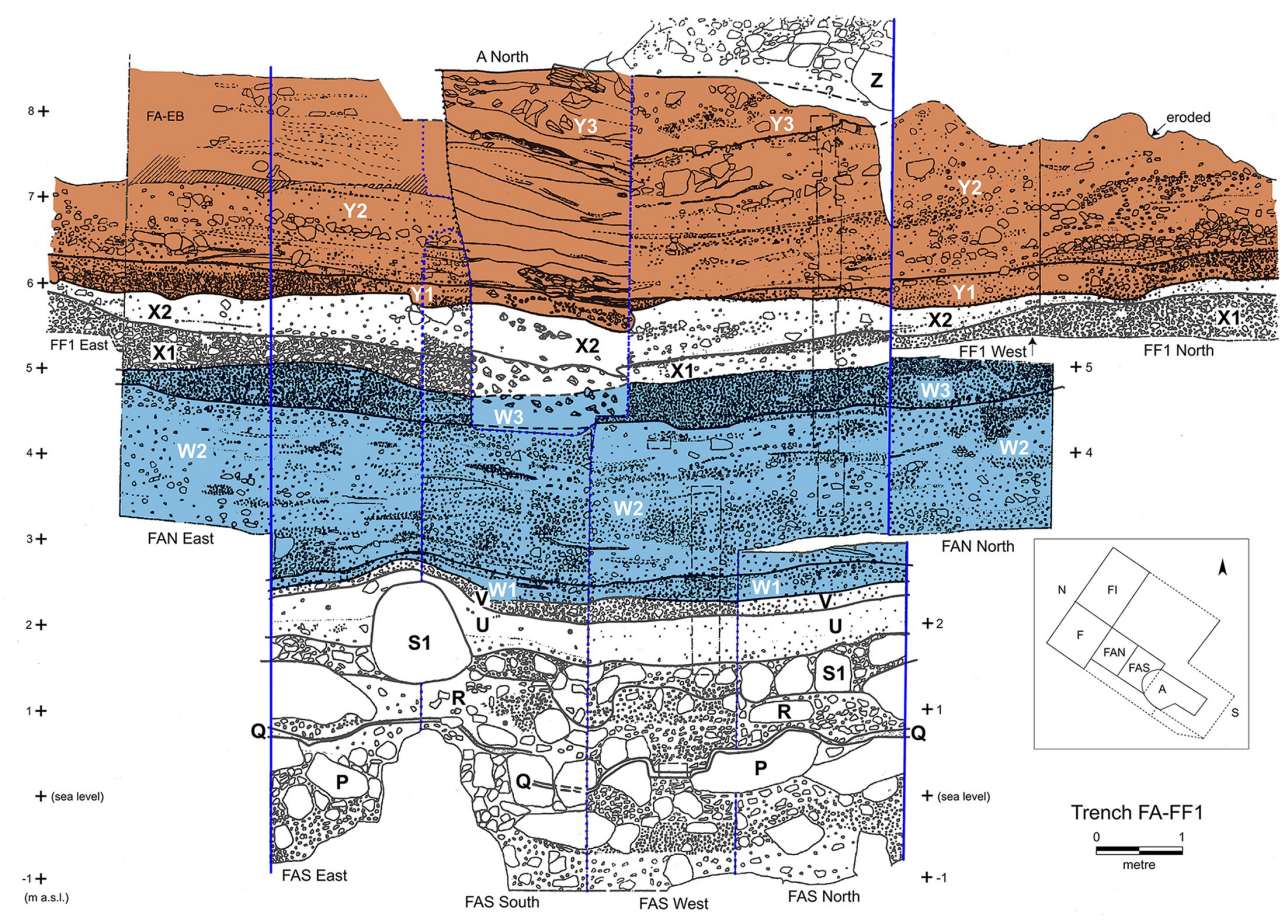
Trench FA-FF1 composite section highlighting key FC strata represented in the anthracological assemblage. Trench FAS sections are demarcated with blue lines (redrawn based on data presented in Farrand 2000 [11]: Plates 8–9 and Fig 4.1).

**Fig 6:**
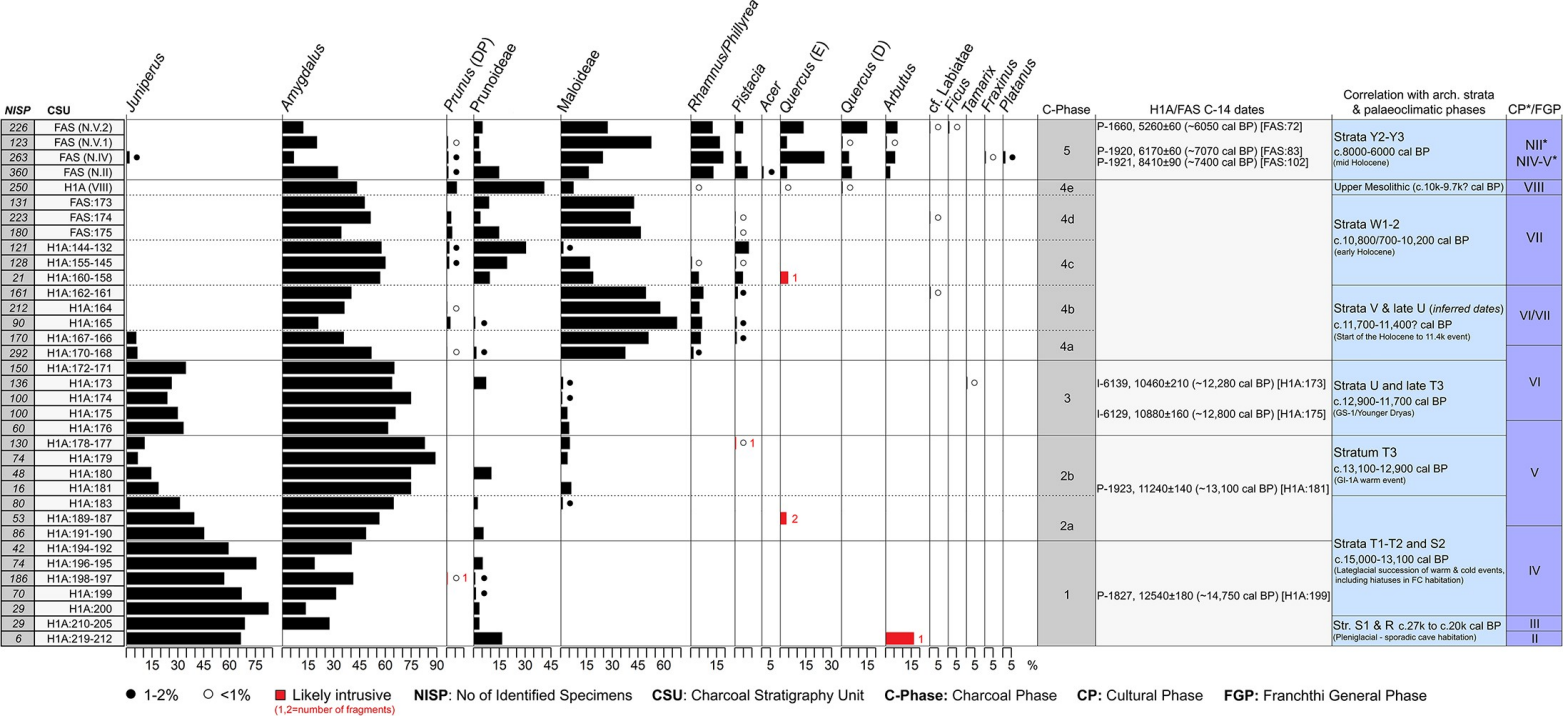
FC anthracological diagram.

92 charcoal samples (1 sample = 1 FC excavation unit) were studied from trenches H1A and FAS (see Figs 3–5) corresponding to a total count of 4835 examined wood charcoal fragments, of which 4453 were botanically identified to genus or family level (S1A Data File). Further data reduction resulted in a population of 4420 botanically identified charcoal fragments spread across 35 Charcoal Stratigraphy Units (CSUs) (S2 and S3 Data Files). CSUs provided the baseline data for the anthracological diagram (Fig 6, S3 Data File; see also the detailed description provided in the Materials and methods section). Five Charcoal Phases (C-Phases 1–5, including seven sub-phases) were identified:

#### C-Phase 1 (~28,000? to ~14,500 cal BP) (including hiatuses)

*Juniperus* (juniper) is the dominant taxon (values ~56%-83%) followed by *Amygdalus* (almond). CSUs H1A:219–212 (all sampled units from stratum R) and H1A:210–205 (all sampled units from stratum S1) produced very low wood charcoal counts (total NISP/number of identified specimens = 36) compared to the CSUs of stratum S2 (H1A:200 to H1A:194–192, with a total NISP = 487). However, as there are no tangible differences in sample composition between strata R/S1 and S2, it is highly likely that the leap in S2 NISP reflects a pattern of more intensive fuel wood use at FC after ~15,000 cal BP rather than an actual shift in local woodland composition and cover.

#### C-Phase 2 (~14,500 to ~12,900 cal BP)

Two sub-phases have been identified: 2a includes the uppermost CSU of stratum S2 (H1A:191–190) and the CSUs of T1-T2 (H1A:189–187, 183). It marks the first notable increase in the proportions of *Amygdalus* (which becomes the dominant taxon) concurrently with a decrease in *Juniperus* (dropping below 45%). The boundary between sub-phases 2a and 2b is characterized by a further drop in *Juniperus* values to <20% in H1A:181 (stratum T3) and was preceded by the first appearance of Maloideae (possibly wild pear) in the FC charcoal sequence, in CSU H1A:183.

#### C-Phase 3 (~12,900 to ~11,700 cal BP)

C-Phase 3 spans the stratigraphically latest CSUs of stratum T3 (H1A:176, 175) and the bulk of stratum U (up to CSU H1A:172–171). Its main characteristic is the resurgence of *Juniperus* values (ranging between ~26%-35%). Although *Amygdalus* is still the dominant taxon, its frequencies are somewhat depressed by comparison to sub-phase 2b. Maloideae are present in low frequencies (~1%-5%) and they disappear at the end of C-Phase 3.

#### C-Phase 4 (~11,700 to ~9500 cal BP)

5 sub-phases (4a-4e) have been identified: 4a marks a radical departure with earlier phases, characterized by the dramatic reduction of *Juniperus* values from ~35% at the end of C-Phase 3 to ~6% at the start of sub-phase 4a. Equally abrupt is the leap in the values of Maloideae (>35%) which now co-dominate charcoal sample composition alongside *Amygdalus*. Another distinguishing feature of the start of C-Phase 4 is the presence, for the first time, of *Rhamnus/Phillyrea* (buckthorn/mock privet) and *Pistacia* (terebinth) in low frequencies. Sub-phase 4b marks the disappearance of *Juniperus* from the charcoal sequence. Maloideae increase further (~50%-68%) while *Phillyrea/Rhamnus* and *Pistacia* remain constant. Sub-phases 4c and 4e include CSUs that are not directly comparable to the rest of the H1A charcoal sequence due to sub-optimal retrieval methods (small-scale bucket flotation). Sub-phase 4d contains 3 CSUs derived from a different trench (FAS:175, 174, 173) which were not stratigraphically contiguous with the part of the H1A charcoal sequence represented in 4a-4c and 4e. It was not possible to establish a more precise stratigraphic correlation between 4d and 4c beyond stating that 4c CSUs H1A:155–145 and H1A:144–132 derived from the same stratum (W2) as 4d CSUs FAS:175, 174, 173. The increasing values of *Prunus* (DP) (wild cherries/plums) in sub-phases 4c-e are also noteworthy. Evergreen and deciduous *Quercus* (oak) also appears in low frequencies for the first time in the uppermost CSUs (H1A:100, 101) of sub-phase 4e (corresponding to stratum X2/Upper Mesolithic).

#### C-Phase 5 (~8100 to ~6000 cal BP)

No wood charcoals were available from layers corresponding to the Final Mesolithic and the Initial-Early Neolithic periods. C-Phase 5 covers the Middle and Late Neolithic periods as represented in trench FAS. Its onset marks several important changes in the FC charcoal sample composition by comparison to the sampled pre-Neolithic phases. Evergreen and deciduous oaks are ubiquitous in the majority of the Middle and Late/Final Neolithic CSUs. Other classic Mediterranean indicator taxa such as *Arbutus* (strawberry tree) and *Acer* (maple) also appear for the first time, while *Rhamnus/Phillyrea* registers higher values compared to C-Phase 4. By contrast, *Amygdalus* frequencies decline. Riparian taxa such as *Fraxinus* (ash) and *Platanus* (plane tree) also appear for the first time in C-Phase 5 in low frequencies.

### Multivariate analysis of dendroanthracological features

**Fig 7:**
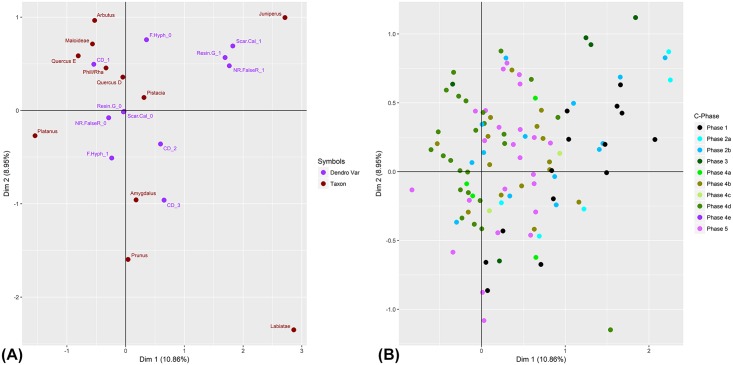
Results of Multiple Correspondence Analysis (MCA) of dendroanthracological features. (A) MCA plot of variables; (B) MCA plot of individuals grouped by Charcoal Phase (C-Phase).

**Fig 8:**
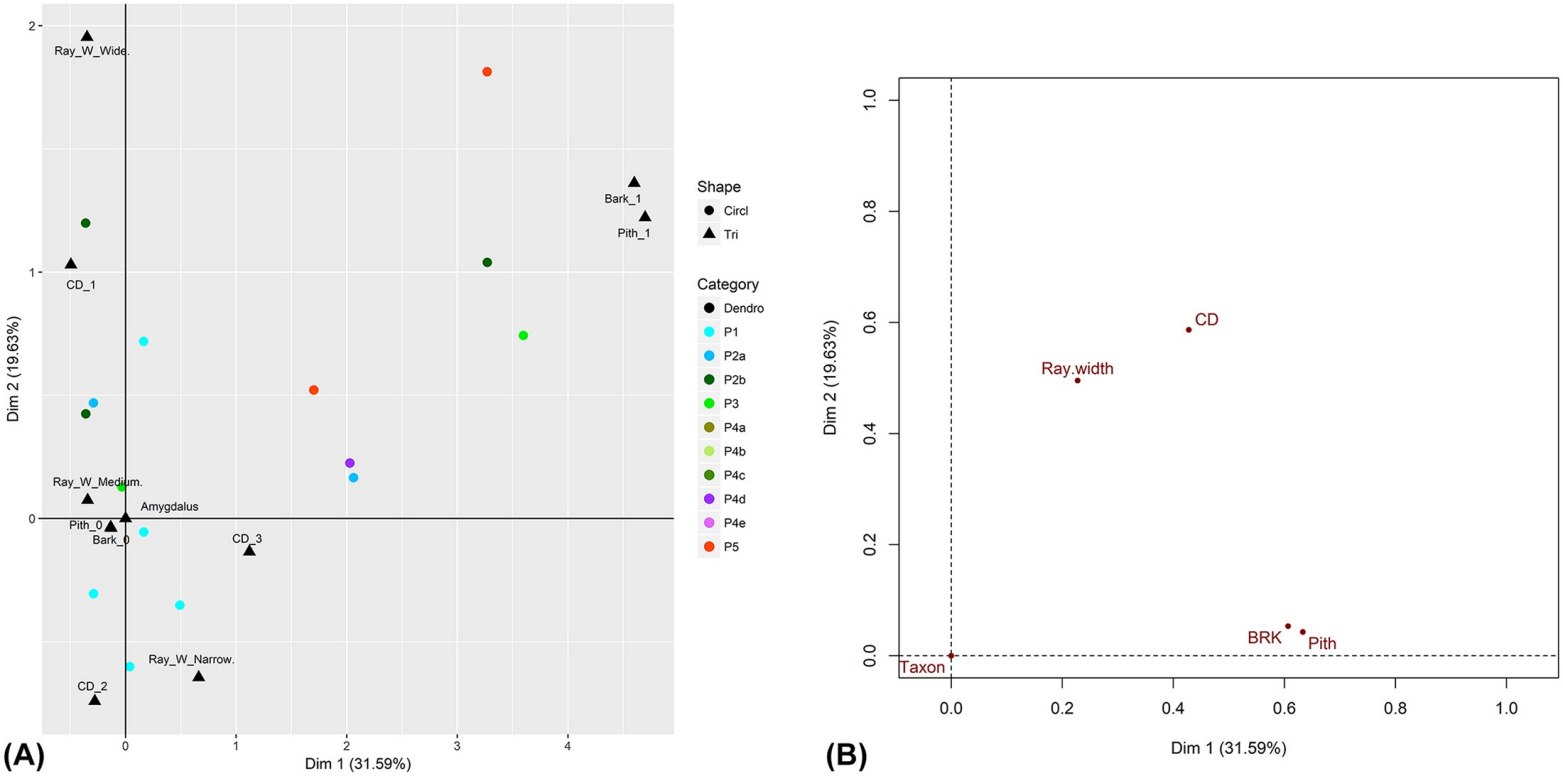
Results of Multiple Correspondence Analysis (MCA) of dendroanthracological features for *Amygdalus*. (A) MCA plot of *Amygdalus* individuals grouped by C-Phase; (B) MCA plot of variables (Taxon = *Amygdalus*).

Dendroanthracological wood anatomical features characteristic of plant growth conditions (curvature degree, false/narrow growth rings, traumatic resin/gum ducts, fungal hyphae, scar/callus tissue) were recorded for 1344 charcoal fragments >4mm that were distributed across C-Phases 1–5. Fig 7A and 7B (S4A and S4B Data File) presents the results of Multiple Correspondence Analysis (MCA) performed on this charcoal fragment population. The plot of variables (Fig 7A) and the plot of individuals grouped by C-Phase (Fig 7B) demonstrate significant overlap in the distribution of dendroanthracological features across phases and between taxa. A clear separation is observed in the clustering of traumatic resin ducts and narrow/false rings with *Juniperus* (dominant in C-Phases 1–3 but absent from C-Phases 4–5) and of large caliber (CD1) charcoal with *Quercus*, *Rhamnus/Phillyrea* (dominant in C-Phase 5) and Maloideae. For Maloideae this pattern is likely to be strongly influenced by the nature of their wood anatomy. Maloideae comprise exclusively diffuse-porous species and are normally characterized by wide annual growth rings. These genetic properties tend to overestimate the representation of large caliber wood. Fig 8A and 8B (S5A and S5B Data File) presents the results of MCA performed on a population of 502 *Amygdalus* fragments >4mm, exploring the co-variation of curvature degree, classes of ray width, and presence of bark and pith. The plot of individuals grouped by C-Phase (Fig 8A) indicates a strong relationship between ray width and caliber: wide and medium ray-width is observed more commonly in large caliber almond charcoals (CD1), whereas narrow ray-width is observed predominantly in medium to small caliber almond charcoals (CD2 and CD3). This relationship is also demonstrated in the plot of variables (Fig 8B). It provides the strongest indication that ray width variation in *Amygdalus* is a function of growth form (i.e., small-diameter round wood vs. large-diameter stem wood) rather than the presence of different species of wild almond. Although it is theoretically possible that the FC *Amygdalus* wood charcoal originated from more than one wild almond species, the MCA results suggest that ray width (a potential species indicator; see discussion in Materials and methods) is predominantly correlating with caliber. Fig 8A also captures a (somewhat weak) temporal trend: *Amygdalus* charcoals of large diameter and ray width are more frequently observed in C-Phases 4 and 5 (Mesolithic-Neolithic), while smaller caliber *Amygdalus* charcoals with narrow rays seem to be better represented in C-Phases 1–2 (Palaeolithic). This suggests that during the Palaeolithic period almond fuel wood derived mostly from smaller shrubs (as opposed to twigs; this is indicated by the lack of a close association of the presence of pith and bark with CD2, CD3 and Palaeolithic samples) while during the Mesolithic-Neolithic larger caliber charcoal including some twigs (= pith & bark) are more frequently observed.

### Sample-by-sample integration of charcoal and seed/fruit data

**Fig 9:**
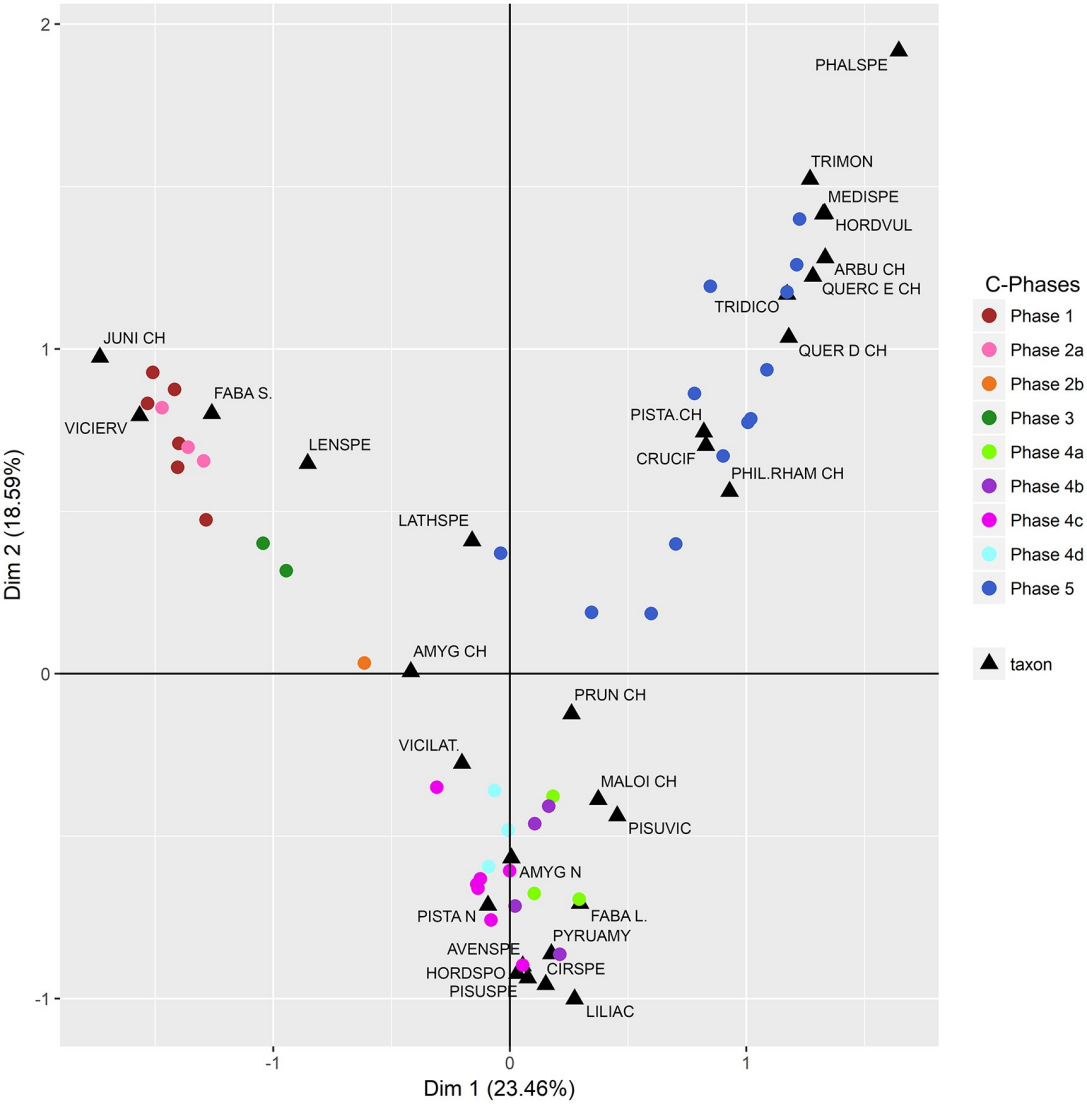
Results of Correspondence Analysis (CA) of charred wood and non-wood botanical taxon counts. CA plot of individuals grouped by C-Phase.

Fig 9 (S6A and S6B Data File) shows the results of Correspondence Analysis (CA) performed on 43 botanical samples spread across C-Phases 1–5, which contained both wood and non-wood (seed/fruit/nut) charred plant remains. The samples of C-Phases 1–3 demonstrate the close association of *Juniperus* charcoal with legume seeds, especially *Vicia ervilia* (bitter vetch), *Lens* (lentil) and small-seeded Fabaceae. *Lathyrus* (pea) seeds and *Amygdalus* charcoal occupy an intermediate position between the Palaeolithic (C-Phases 1–3) and the Mesolithic (C-Phase 4) samples. C-Phase 4 samples indicate the close association of *Prunus* and Maloideae charcoal with *Pistacia* and *Amygdalus* nuts, *Pyrus* (wild pear) fruits, *Hordeum vulgare spontaneum* (wild-type barley), *Avena* (oats) and legumes (large-seeded Fabaceae, *Pisum/*pea, Viciae), and *Cirsium* and Liliaceae seeds. The Neolithic (C-Phase 5) samples point to the close association of *Quercus*, *Pistacia*, *Arbutus* and *Phillyrea/Rhamnus* wood charcoal with domesticated cereal crop species such as emmer (*Triticum dicoccum*) and einkorn wheat (*T*. *monococcum*), 2-row hulled barley (*Hordeum vulgare*), plus Cruciferae (Brassicaceae) and the small-seeded grass *Phalaris*.

### Meta-analysis of the FC non-wood archaeobotanical dataset

**Fig 10:**
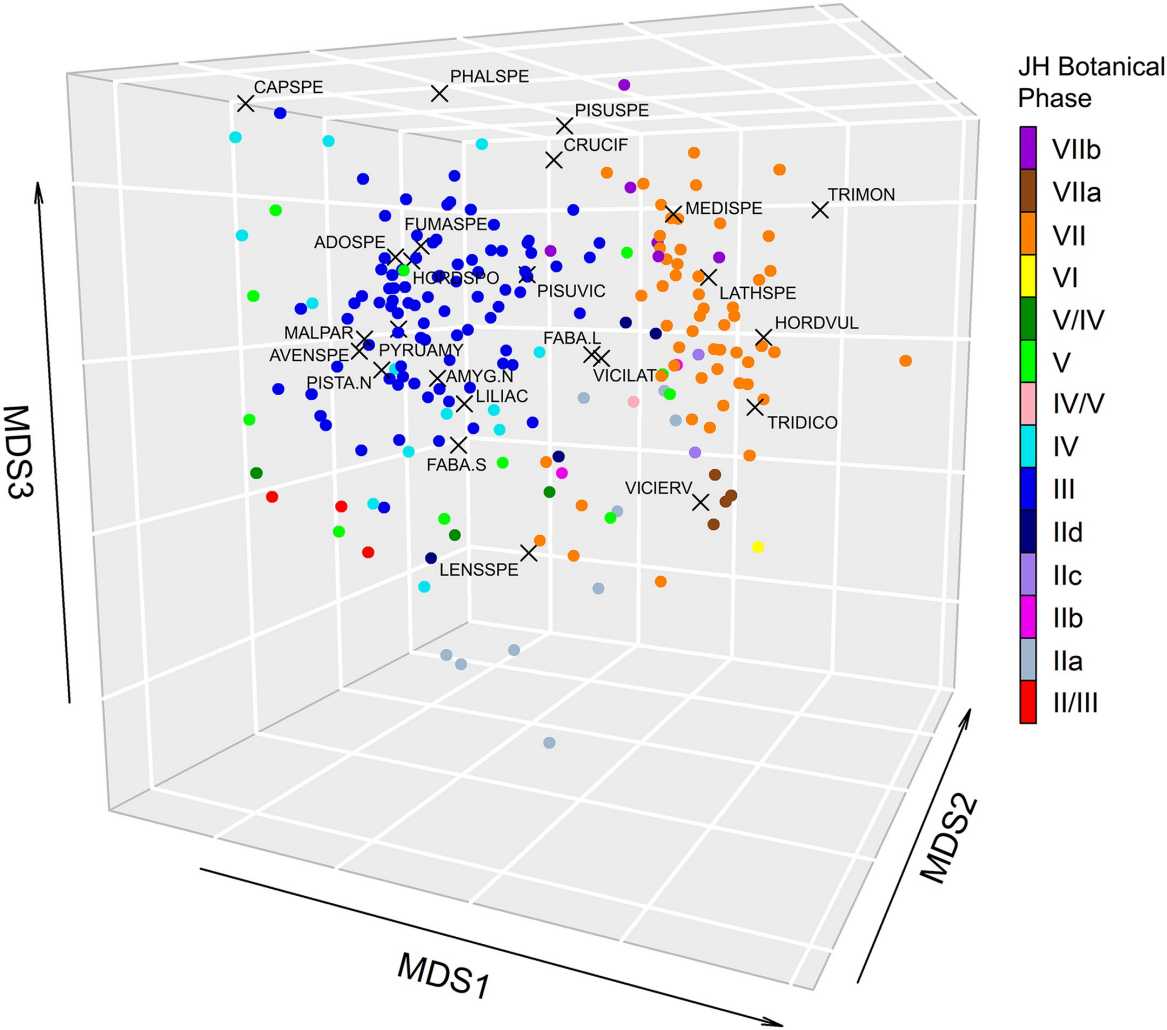
Results of 3D Non-Metric Multi-Dimensional Scaling (NMDS) of non-wood botanical taxon counts. NMDS ordination was undertaken on an abundance per species matrix.

**Fig 11:**
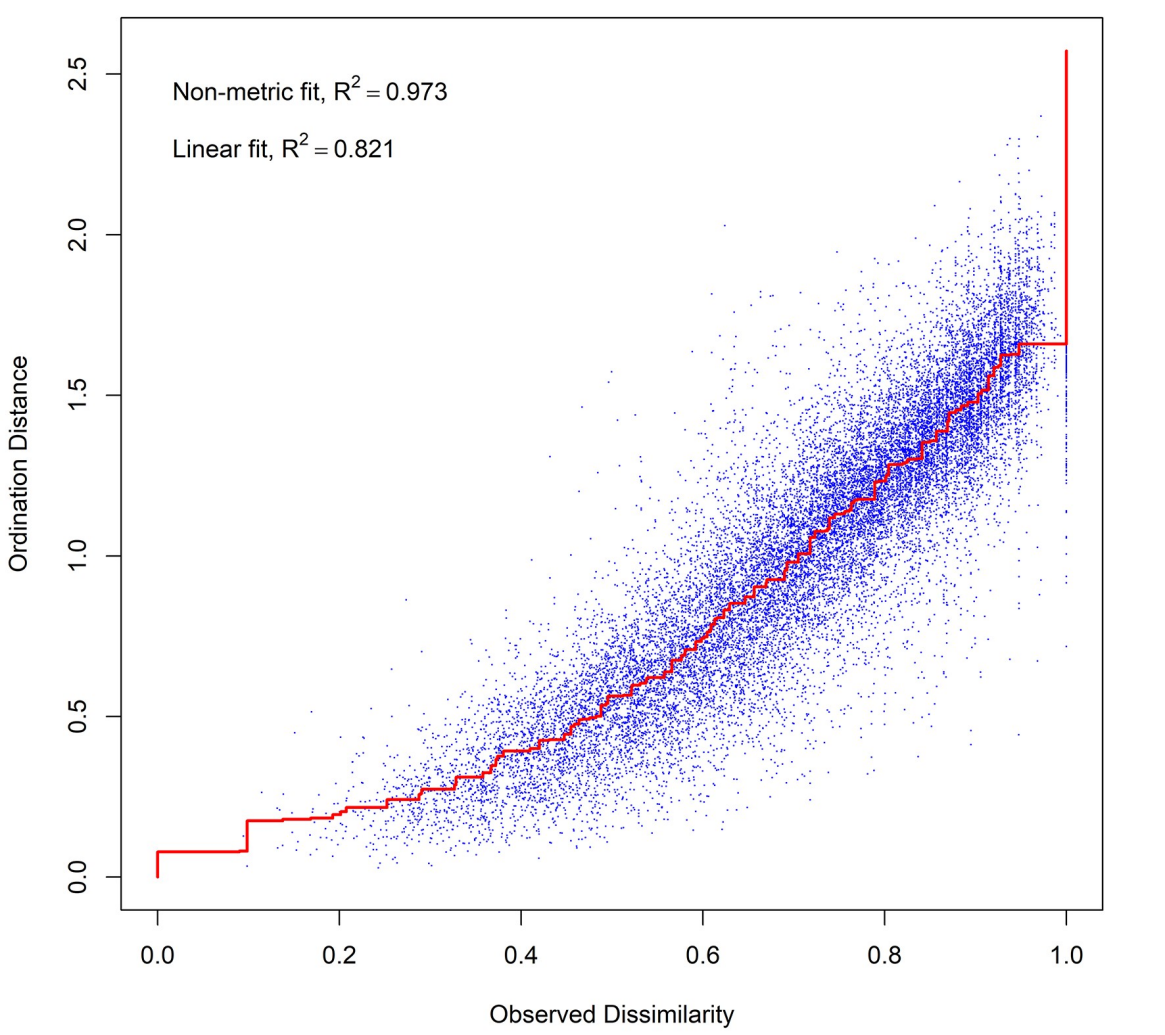
Shepard stress plot. Relationship between NMDS ordination distance and original observed distance.

Figs 10 and 11 (S7 Data File) present the results of 3D Nonmetric Multi-Dimensional Scaling (NMDS) performed on 199 botanical samples covering the entire FC sequence that was sampled by flotation in the 1970s (trenches H1A, H1B, FAS and FAN). The Shepard stress plot (Fig 11) (stress value = 0.165) indicates a satisfactory level of representation of the main compositional gradients in the dataset. In Fig 10 samples are colour-coded following Hansen’s botanical zones (JH Zones I-VII; see also S1B-1 Data File) to facilitate comparisons with the non-wood archaeobotanical findings published in the 1990s [14]. The results demonstrate clear temporal patterning in sample composition. The clearest distinction is evidenced, as expected, between the Neolithic (JH Zones VII-VI) and the Palaeolithic and Mesolithic samples (JH Zones IIa-IId-III-IV-V). *Lens* is both ubiquitous and abundant in the latter. Other large-seeded legumes (*Lathyrus*, *Vicia ervilia* and large-seeded Fabaceae) appear consistently, if sporadically, throughout the FC sequence. The legume spectrum expands in the Mesolithic period (JH Zone III) with the addition of *Pisum*. *Amygdalus*, *Pistacia* and *Pyrus* are also more frequent and ubiquitous in the Mesolithic samples, being closely associated with *Avena*, *Hordeum vulgare spontaneum* and a limited range of wild seed taxa (e.g., *Malva*, *Adonis*, *Fumaria*, Liliaceae, small-seeded Fabaceae). Wild-type cereal taxa disappear in the Neolithic (JH Zones VI-VII). Most Neolithic samples cluster around the domesticated cereal crops (emmer, 2-row hulled barley and, in the later Neolithic phases, einkorn) plus *Medicago* (medick) and, to a lesser extent, *Vicia ervilia* and *Lathyrus*. The abundant presence of *Medicago* (a small-seeded legume) in the Neolithic samples may indicate increasing agropastoral impacts on the local landscape. *Medicago* is a caprine forage species and could have been incorporated into sample composition as a weed of cultivation, a fodder crop and/or a component of caprine pastures. By contrast, *Vicia ervilia* and *Lathyrus* are present in much lower frequencies. The consistently low frequencies and ubiquity of the remaining large-seeded legumes (including *Lens*) in the Neolithic samples may point to their potential incorporation into the archaeobotanical record as elements of background vegetation rather than as cultivars on their own. Although average lentil seed size increased somewhat in the Initial Neolithic, the observed variation as reported by Hansen [14] still falls within the size range of wild-type *Lens* spp. Furthermore, in later Neolithic phases this trend was reversed with average seed size being directly comparable to that of Mesolithic specimens [14]. Another noteworthy characteristic of the FC non-wood botanical assemblage is the apparent irregularity in the frequency and density distributions of the small-seeded grasses and legumes, which persists across all phases. This is undoubtedly the result of the use in the 1970s of a very large (by modern standards) flotation mesh size (~1.5mm instead of the presently recommended 0.25mm) which could not retain most small-seeded taxa that were potentially preserved in the botanical samples (and almost certainly retained some seeds erratically, e.g., as clumps attached to larger charred plant items). A significant implication of this inherent, non-rectifiable recovery bias is that it precludes exploring with the presently available FC non-wood archaeobotanical dataset the hypothesis that predomestication cultivation (see overview in [21] and references therein) was practiced at FC during the pre-Neolithic periods.

## Discussion

### Vegetation history, climate change and anthropogenic impacts

Early Lateglacial vegetation in the environs of FC comprised open *Juniperus-Amygdalus* woodland. *Juniperus* was the dominant taxon during the timespan represented by strata R, S1 and the greater part of S2 (C-Phase 1) growing under cold and dry conditions, as suggested by the frequent presence of environmental stress indicators such as narrow/false rings and traumatic resin ducts. Junipers are shade-intolerant and thrive in well-drained, nutrient-poor soils. For this reason, they are commonly associated with pioneer plant communities that are characteristic of early postglacial vegetation development. Although it is not possible to identify junipers to species level based on the wood anatomical structure of charcoals alone, it is noteworthy that none of the Mediterranean thermophilous species presently distributed in southern Greece can tolerate cold and arid climes. It is therefore highly unlikely that the FC juniper charcoals could have derived from a Mediterranean lowland species such as *J*. *phoenicea* that abounds in the area today. Another species native to the Peloponnese that is both frost- and drought-tolerant (but intolerant of heat) is *J*. *drupacea*, reported in the literature to form geographically disjunct communities in montane woodlands in Laconia and Arcadia at altitudes of 800-1700m a.s.l. [26]. *J*. *drupacea* survived in montane refugia during the late Pleistocene migrating downwards during cold spells and upwards in warmer periods [27]. Other species whose late Pleistocene distributions might have exhibited similar altitudinal migration patterns include dwarf common juniper varieties such as *J*. *nana* and *J*. *hemisphaerica*, presently found in montane cold grassland habitats in the Peloponnese [28]. The hypothesis for the presence of a cold- and drought-tolerant *Juniperus* species at FC that is currently extinct from this area finds additional support in the fact that juniper charcoal frequencies decrease through time concurrently with climatic amelioration. *Juniperus* declines dramatically in sub-phase 4a and is completely absent from sub-phases 4b-e. Only 4 juniper charcoal fragments were found in the CSUs of Neolithic C-Phase 5. These (if not intrusive from late prehistoric and/or historic periods of cave use) could have derived from a thermophilous species growing near FC during the Neolithic. However, considering also the high charcoal counts obtained from C-Phase 5, this is improbable. Overall, the status of *Juniperus* in the FC charcoal sequence suggests that the present-day abundance of the thermophilous *J*. *phoenicea* in the local vegetation is a late Holocene development, very likely post-dating the Neolithic period.

*Amygdalus* (including the xerophytic wild almond *A*. *webbii* that is widely distributed in southern mainland Greece) is another taxon that is well adapted to nutrient-poor soils. Being equally well suited to cold and warm climes, and highly resilient to competition by grasses and herbs for seasonally deficient ground moisture resources [29] *Amygdalus* expanded rapidly on the FC coastal plain during the temperature and precipitation seesaw of the Lateglacial (~15,000–12,900 cal BP) eventually replacing cold-loving *Juniperus* as the dominant species. The same trend is observed during the Younger Dryas (C-Phase 3; ~12,900–11,700 cal BP) that witnessed only a transient restoration of *Juniperus* charcoal frequencies to values approximating those of sub-phase 2a (early Lateglacial). Even under the Younger Dryas cold and arid regime, *Juniperus* did not replace *Amygdalus* as the dominant species (see Fig 6). It is possible that the vegetation impacts of increasing climatic aridity were, at least to some extent, mitigated by the proximity of the coastline. At the same time, however, the rapid increase of *Juniperus* values in the uppermost CSUs of stratum T3 points to a rather quick onset of quasi-continental conditions (including cold winter and spring seasons, and relatively short summer seasons) to which the cold-loving junipers are well-adapted. It is also likely that a net decrease in precipitation favored both the grasses and the more cold-tolerant large-seeded legumes at the expense of the already sparse arboreal cover, due to the greater capacity of annual plants to compete effectively with woody perennials for limited ground moisture resources. A comparable dynamic vegetation response pattern has been observed in the Mediterranean littoral of the southern Levant, where grasses persisted during the Younger Dryas unlike their retreat from inland Irano-Turanian ecoregions [21].

**Fig 12:**
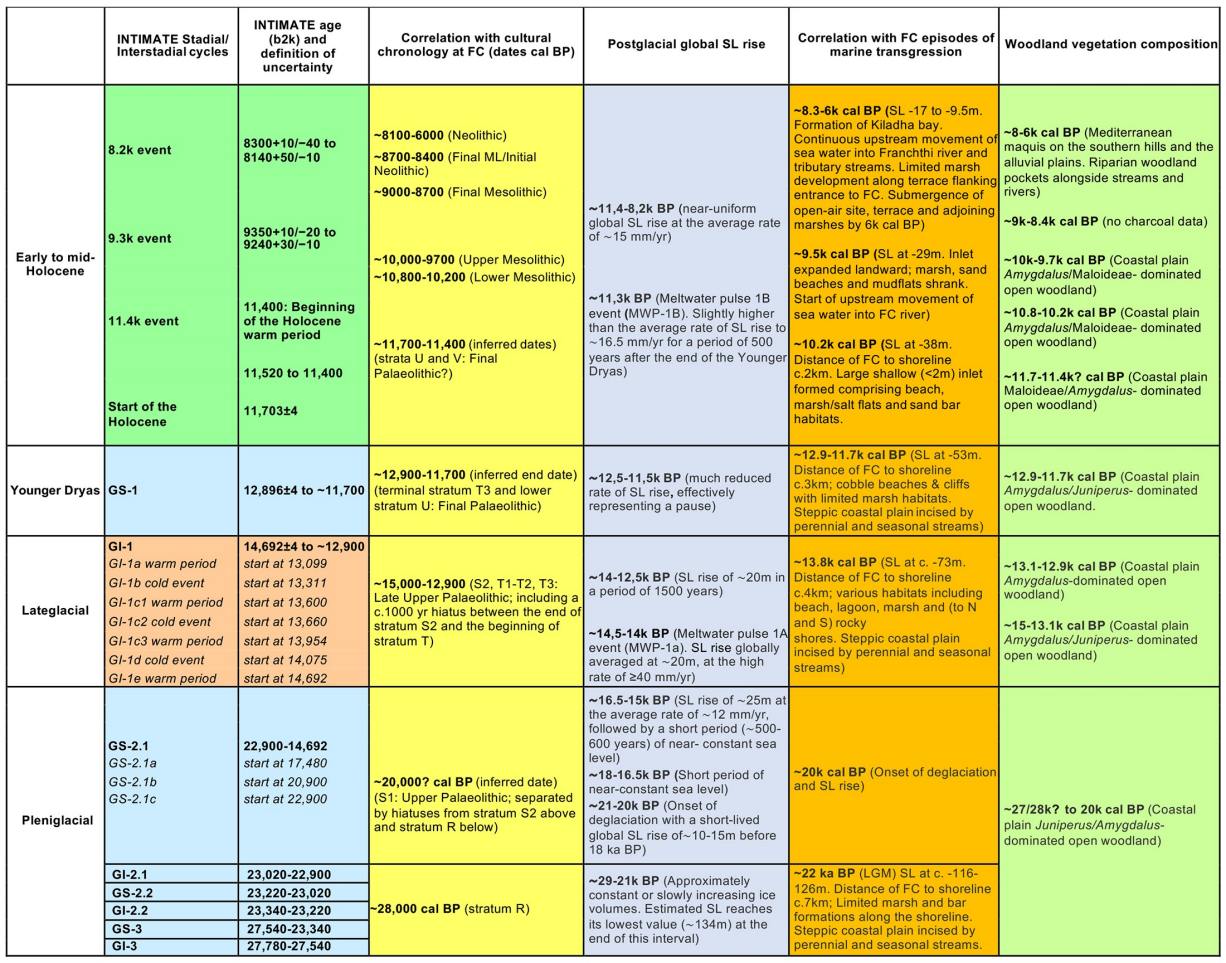
Correlation of reconstructed woodland composition with sea-level rise, FC cultural chronology and INTIMATE Greenland ice core chronostratigraphy. Global ice core data and chronology retrieved from [30], global sea-level rise data from [1], FC regional sea-level and shoreline models from [15–16, 32] and archaeological chronology data from [11, 20, 24].

Unlike other categories of archaeological materials, which were apparently strongly influenced by cultural choices and the periodicity of occupation at FC [24] the charcoal stratigraphy is very closely aligned with the local and regional diachronic palaeoenvironmental trends that affected woodland composition and ecology in the site environs. The fluctuations in the frequencies of *Juniperus*, *Amygdalus* and Maloideae through the late Upper/Final Palaeolithic segment of the FC anthracological sequence reveal sharp boundaries between charcoal phases 2b, 3 and 4, which correspond closely to the GI-1a warm period, the Younger Dryas and the start of Holocene respectively (Figs 6 and 12, S3 Data File). The onset of sub-phase 2b (stratum T3) is marked by the reduction of *Juniperus* and a concurrent increase of *Amygdalus*. The start of C-Phase 3 is marked by the rapid increase in *Juniperus* within the uppermost units of T3. Sub-phase 4a is marked by the dramatic drop of *Juniperus* alongside a reduction of *Amygdalus* and an equally dramatic peak in Maloideae values. In sub-phase 4b *Juniperus* disappears. This temporal trend in *Juniperus* charcoal frequencies is derived from stratigraphically contiguous flotation samples, thus permitting assigning with a high degree of confidence the otherwise undated sub-phase 4a to the Younger Dryas termination and the start of the Holocene at ~11,700 cal BP. The abrupt drop of *Juniperus* charcoal frequencies observed in H1A:170–166 directly overlies the sampled units in strata U and T3, which recorded high *Juniperus* values and are securely radiocarbon dated to the Younger Dryas. The continuous, high-resolution record of charcoal deposition obtained from this segment of the H1A charcoal stratigraphy permits treating the *Juniperus* charcoal curve as a reliable palaeoecological proxy for assigning the upper end of stratum U (H1A:170–169) and the basal units of stratum V (H1A:168–166) to the YD termination and the start of the Holocene. In turn, dating this stratigraphic boundary to ~11,700 cal BP agrees well with both the Greenland ice core stratigraphy and other high-resolution palaeoecological sequences available from the Eastern Mediterranean [30–31].

An important implication of linking the strata U/V boundary to the start of the Holocene is that the sedimentary characteristics of stratum V as reported in [11] (i.e., abundant white rock fragments 5–10 cm with little dark yellowish-brown matrix, including some charcoals and snail shells) are likely to reflect the impact of a prominent episode of climatic instability known as the Preboreal Oscillation (PBO-11.4k event). Based on the Greenland ice core isotopic records, the PBO initially manifested as a short warm episode that started within the first 50 years after the end of the Younger Dryas, which was then followed by ~100 years of cooling and ended with a rapid reversal to warm conditions at ~11,300 cal BP [30, 33]. The extent to which such rapid climatic reversals could have exerted lasting (hence archaeobotanically detectable) impacts on the local vegetation is debatable. There is, however, little doubt that they affected the seasonal availability of edible plant and faunal resources, and that they occurred at timescales that were sufficiently short to have influenced directly people’s experiences and perceptions of the changing climate and the environment. The PBO might thus explain, at least in part, the depositional hiatus claimed for stratum V [11–12]. Future radiocarbon dates obtained from strata U and V may help resolve the uncertainty surrounding their chronology, including whether there was a catholic hiatus in archaeological deposition at FC, and probe further the palaeoecological significance of the *Juniperus* charcoal curve in H1A.

The bulk of C-Phase 4 (sub-phases 4c-e) comprises samples originating in two different trenches (H1A, FAS) which were not stratigraphically contiguous, and (except for FAS:175, 174, 173) did not derive from excavation units sampled by large-scale tank flotation. Thus, their utility for reconstructing changes in woodland composition within C-Phase 4 is limited. The CSUs of sub-phases 4c-d are associated with strata W1-2 (Lower Mesolithic: mid-late 11^th^ millennium cal BP) while sub-phase 4e represents Upper Mesolithic deposits (stratum X2: early 10^th^ millennium cal BP). Sub-phase 4d CSUs (FAS:175, 174, 173) display similarly high Maloideae values to the CSUs of 4a-b (which were also processed by large-scale tank flotation). On the other hand, *Amygdalus* appears to dominate the CSUs of sub-phase 4c that were sampled by small-scale bucket flotation. However, despite the high charcoal counts obtained from sub-phase 4d, *Pistacia* is markedly under-represented in it (and *Rhamnus/Phillyrea* altogether absent) and in 4e (Upper Mesolithic), in marked contrast to the comparatively high charcoal counts recorded for both taxa in sub-phases 4a-c. A potential explanation for these discrepancies is that they were caused by unknown taphonomic factors affecting the sampled excavation units, which render problematic the interpretation of the shifts observed in charcoal sample composition during the timespan represented by sub-phases 4c-e.

Overall, the integrated wood and non-wood botanical data indicate that the Mesolithic vegetation is qualitatively distinguished from that of the preceding Palaeolithic phases by the dominance of the rainfall-dependent Maloideae (most likely representing *Pyrus*/wild pear) alongside the more heat-tolerant *Amygdalus*. Wild pears and almond shrubs grew in association with *Avena/Hordeum*-dominated grasslands, which expanded rapidly on the coastal plains in response to the abrupt temperature and precipitation increases that characterized the start of the Holocene across the Eastern Mediterranean [31]. Another indicator of the prevalence of a more positive moisture balance during the Mesolithic period is the increasing presence of *Prunus*. The dendroanthracological data demonstrate the prevalence of small-caliber round wood in the Palaeolithic and Mesolithic periods, alongside the frequent incidence of fungal hyphae on *Amygdalus* charcoals, which points to the regular collection of dieback deadwood from almond shrubs growing in the FC hinterland. In the Palaeolithic, the prevalence of shrub forms was likely caused by the prevailing cold and arid climes. In the Mesolithic, although the dendroanthracological evidence points to improved growth conditions for almonds, shrub forms probably persisted as an adaptive strategy of perennial woody plants to intensified competition with annual grasses for seasonally deficient ground moisture [29]. Independent palaeoclimatic archives in the Eastern Mediterranean also indicate heightened climatic seasonality in the first two millennia of the Holocene (characterized by hot and arid summers and wet winters favoring the expansion of grasslands) [21].

The absence of evergreen and deciduous oak charcoal (*Quercus*) from both the Palaeolithic and the Mesolithic phases represents perhaps the most important indicator of the ecological distinctiveness of the southern Argolid coastal shelf woodland-grassland biome, especially when considering the fact that oaks were present in the neighboring cave sites of Kefalari and Klissoura (both located in more humid inland areas; see Fig 1A and 1B) in the late Upper Palaeolithic [8]. By contrast, at FC *Quercus* charcoals appear for the first time in very low frequencies in the uppermost units (H1A:100, 101) of stratum X2/charcoal sub-phase 4e (dated to the Upper Mesolithic; early 10^th^ millennium cal BP [20, 24]). Even this date is however questionable, as their presence in the uppermost H1A Upper Mesolithic units might equally represent intrusions from Neolithic layers, which in trench H1A lay directly atop the Upper Mesolithic stratum X2 (Fig 4).

Altogether, the integrated FC wood and non-wood botanical data offer strong support to the hypothesis that *Amygdalus* and Maloideae fuel wood was predominantly collected from the coastal plain, where these taxa formed sparse, open woodlands with an understorey rich in wild cereals and legumes. In turn, these woodlands differed in their botanical composition from the Mediterranean-type woodland habitats previously proposed in the literature [14, 17, 24]. Classic Mediterranean indicator species such as evergreen and deciduous *Quercus*, *Arbutus* and *Acer* were absent, while others (*Rhamnus/Phillyrea*, *Pistacia*) were very infrequently used as firewood. The fact that *Pistacia* nuts were intensively collected during the Mesolithic period suggests that terebinth stands might have grown at some distance from the coastal zone, and that their nuts were selectively harvested and brought back to the site. Alternatively, and more plausibly, it may indicate that *Pistacia* grew in moister localities on the coastal plain (e.g., near springs or other moist grassland habitats alongside *Prunus*) where its stands were protected from over-cutting and managed for their nut crop.

C-Phase 5 represents a clear break in the charcoal stratigraphy. Its CSUs (trench FAS, strata Y2-3) (see Figs 5 and 6) date to the Neolithic (~8000–6000 cal BP). They are dominated by Mediterranean taxa including evergreen and deciduous oaks (*Quercus*) growing alongside *Amygdalus*, Maloideae, *Acer* and *Arbutus* and riparian trees such *Fraxinus* and *Platanus*. *Pistacia* charcoal is also regularly present. In contrast to earlier periods, plant-food species comprise predominantly domesticated cereal crops without significant inputs by nuts gathered from the wild (*Pistacia*, *Amygdalus*). Large caliber charcoal specimens of all taxa are notably ubiquitous in the Neolithic CSUs. This suggests a greater incidence of maquis woodland clearance during this period, possibly in association with cereal cultivation.

### Correlation of shifts in plant palaeohabitats with SLR

**Fig 13:**
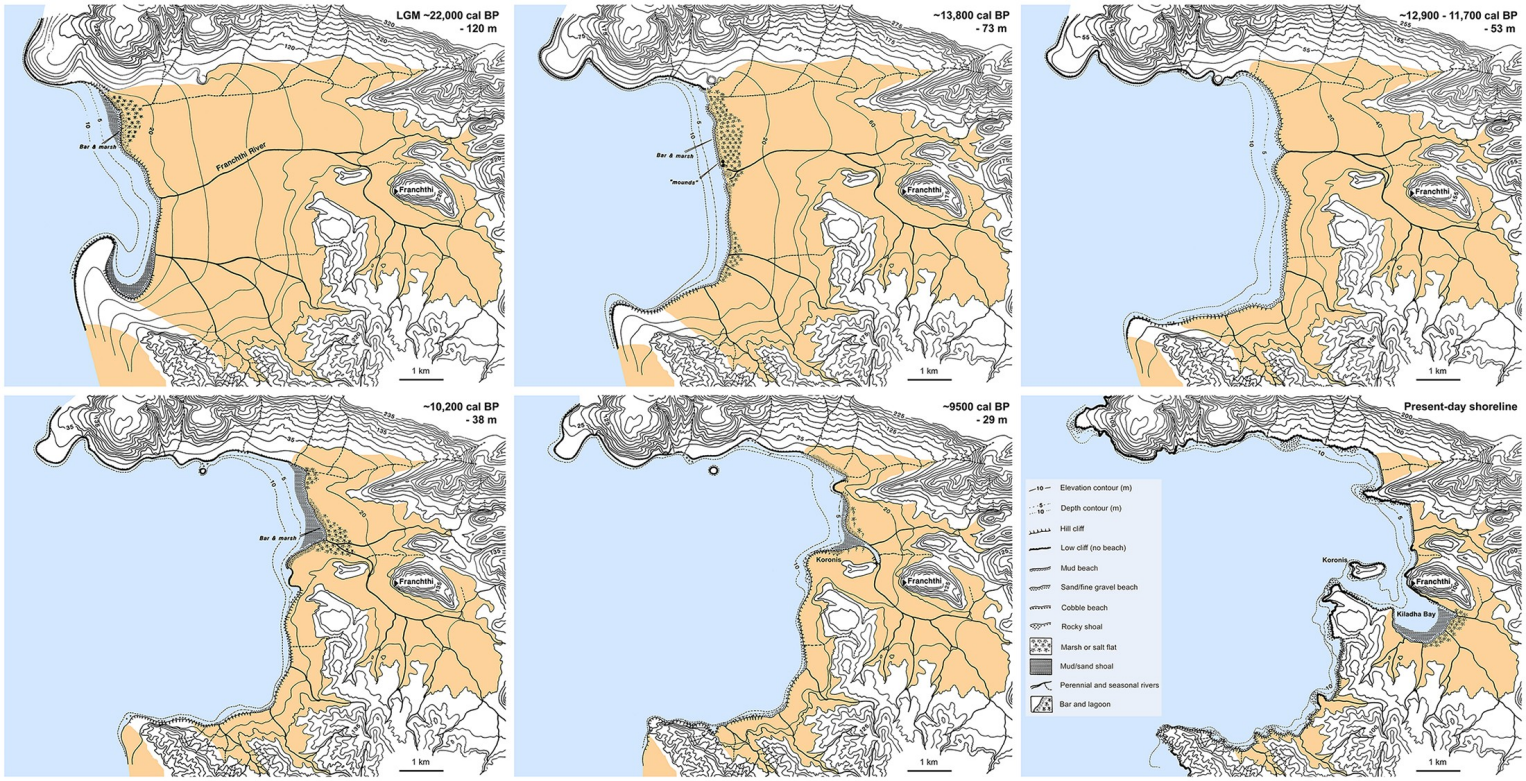
Evolution of FC shoreline and modelled landscape impacts of postglacial sea-level rise. Redrawn based on data presented in Van Andel and Sutton 1987 [15]: Figs 6 and 12–17.

**Fig 14:**
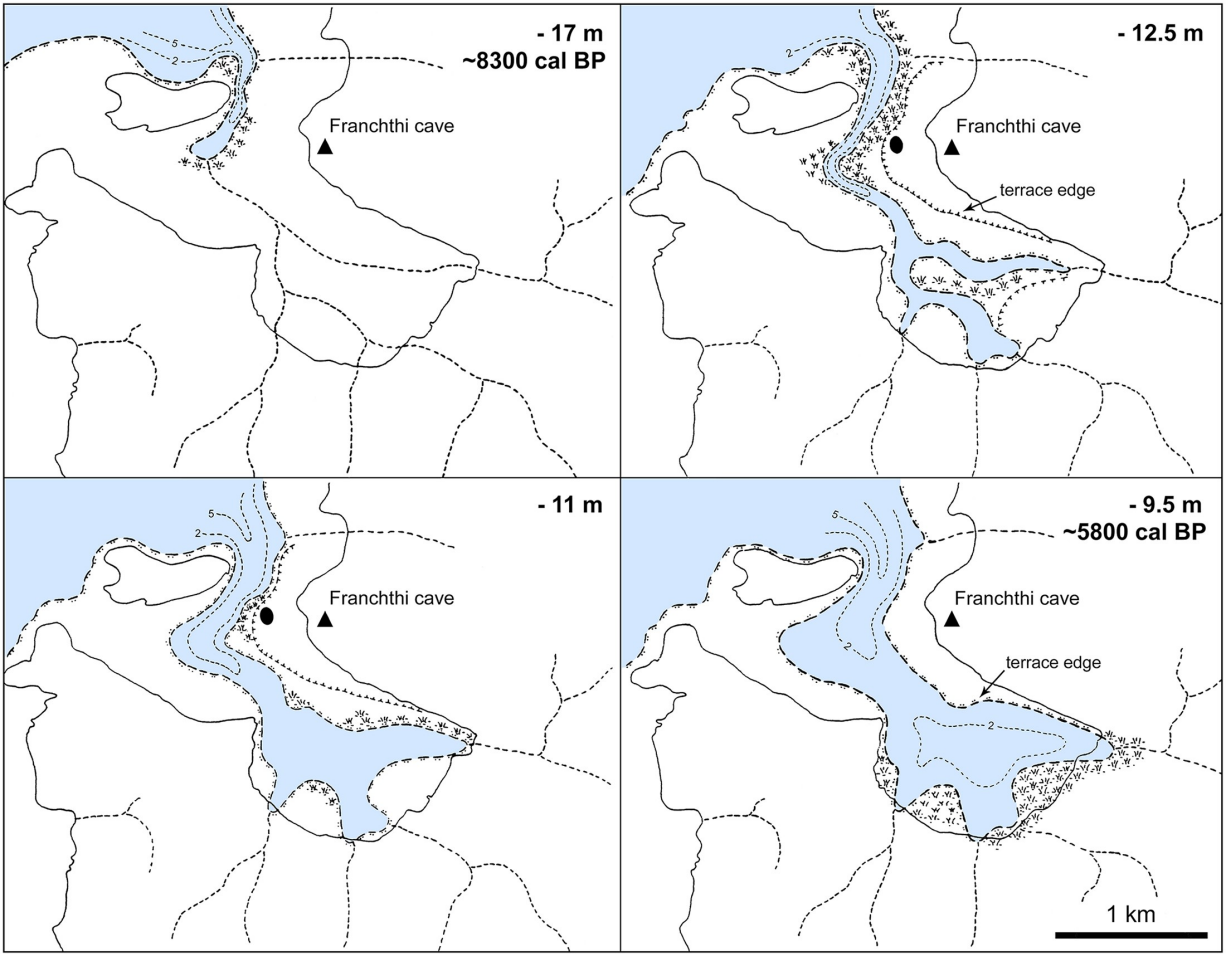
Transgression of sea into the Kiladha bay ~8300–5800 cal BP. Redrawn based on data presented in Van Andel and Sutton 1987 [15]: Fig 21. Black dot denotes location of FC seaside (Paralia) open-air Neolithic site.

**Fig 15:**
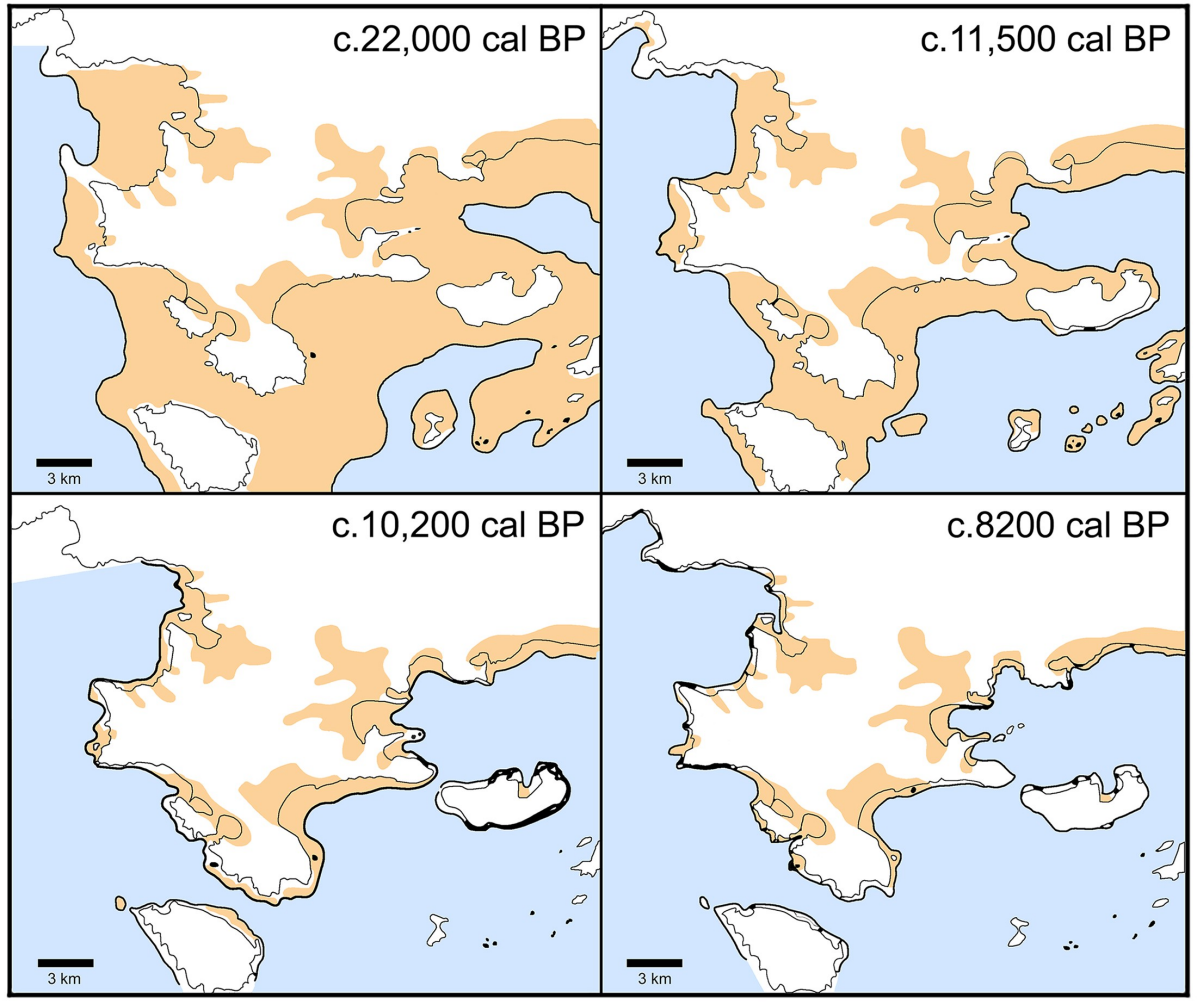
Evolution of southern Argolid peninsula shoreline and modelled landscape impacts of postglacial sea-level rise. Redrawn based on data presented in [32].

Figs 13 and 14 represent the evolution of the FC coastline as reconstructed from the Last Glacial Maximum to the end of the Neolithic period [15–16]. The available evidence points to a high degree of chronological convergence between the charcoal stratigraphy as a proxy of palaeovegetation change, the global palaeoclimatic records, and the available SLR models (see also Fig 12). During the Lateglacial and up until the start of the Holocene at ~11,700 cal BP the exposed coastal shelf formed a dominant feature of the FC landscape. It was rapidly colonized first by *Juniperus*, followed by *Amygdalus* alongside annual legumes and grasses. After approximately a millennium of nearly stable coastal conditions during the Younger Dryas, SLR resumed at the start of the Holocene, albeit at much reduced rates by comparison to the Lateglacial. The abrupt increase in temperature and precipitation that marked the onset of the Holocene across the Eastern Mediterranean was accompanied by the dramatic expansion of grasslands [21, 31]. This is also clearly reflected in the FC Mesolithic archaeobotanical samples that are overwhelmingly dominated by almond and terebinth nuts, wild-type oats, barley and lentils. The integrated charcoal and non-wood botanical data indicate that the coastal shelf sustained dense and highly productive stands of grass-woodland vegetation, even though by the end of the 11^th^ millennium cal BP it had lost approximately 2/3 of its LGM surface area to SLR (Figs 13 and 15). This was the case until ~10,200 cal BP and the end of the Lower Mesolithic, and it was probably a major contributing factor to the intensification of plant-food use observed at FC during this period.

The available evidence suggests that the first signs of palaeoenvironmental pressures on traditionally used plant resources were manifested during the first half of the 10^th^ millennium cal BP. By ~9500 cal BP, continuous (if slow-paced) SLR had brought about the landward expansion of a shallow seawater inlet, and the start of the inundation of the deeply incised Franchthi River valley near Koronis islet in the immediate proximity of FC [15] (Figs 13 and 14). The Upper Mesolithic charcoal data (sub-phase 4e) do not paint a picture of substantial changes in fuel wood species diversity and availability during this period. It appears that most firewood was still collected from pockets of Maloideae-*Amygdalus* woodland that persisted on parts of the increasingly narrowing coastal shelf, or (more likely) the banks of the Franchthi River, the FC terrace and the colluvial slopes abutting the FC promontory. The non-wood botanical evidence indicates very limited plant-food use during this period, which is distinguished by an emphasis on tuna fishing [14, 18]. It is possible that, apart from the increasing inundation of the coastal shelf by SLR, the increasing salinization of the Franchthi River due to rising sea-levels had also began affecting the availability of wild cereal and legume stands on the FC terrace and the riverine floodplain alluvium that formed a dominant feature of the local landscape during the early Holocene [34–35].

Charcoal data from the Final Mesolithic and the Initial-Early Neolithic periods (~9000–8300 cal BP) are lacking from the assemblage available for analysis. For the earlier part of this period, the non-wood botanical data indicate a marked reduction in charred plant densities, while wild oats and barley gradually disappear from the botanical assemblage [14]. *Amygdalus* and *Pistacia* nuts also decrease markedly. The first introduction of domesticated cereal crops to FC is dated to ~8700 cal BP [14, 20]. By 8200 cal BP the coastal shelf had mostly disappeared under SLR in the southern Argolid peninsula (Figs 13–15). Towards the end of the 9^th^ millennium cal BP the sea inlet had broken into the Kiladha bay and seawater continued moving upstream the Franchthi River and its tributaries (Fig 14) [15–16]. Anthracological data are again available from the beginning of the 8^th^ millennium cal BP (Middle Neolithic). They clearly point to the radical reconfiguration of the landscapes of the coastal zone, with Neolithic fuel wood gathering and cereal crop cultivation targeting the Mediterranean maquis and riparian vegetation growing on the hill zone and the alluvial plains that were by that time proximate to the coastline.

## Conclusions

The first systematic application of anthracology at FC has revealed unambiguous differences in the nature and ecology of the vegetation environments proximate to the site during the Palaeolithic, Mesolithic and Neolithic periods, which are closely related to climate change, and the rate and pace of the submergence of the coastal shelf by postglacial SLR. During the Lateglacial and the early Holocene fuel wood and plant-food procurement targeted the open grassland-woodland biome that dominated the still exposed coastal shelf of the southern Argolid peninsula. Late Palaeolithic and Mesolithic plant exploitation focused on the intensive harvesting of almonds (which also provided the bulk of fuel wood) and terebinth nuts supplemented by large-seeded annuals including oats, barley and lentils. This plant resource spectrum (with its emphasis on protein-rich nuts and legumes, and the reliance on almond wood as fuel) overlaps significantly with those known from a majority of late Pleistocene and early Holocene habitation sites located in the semi-arid steppe woodlands of continental Southwest Asia [21–22, 29]. In turn, such similarities point to the convergent evolution of pre-agricultural plant resource choice and exploitation strategies in Southwest Asia and the southern Argolid coastal shelf, due to the opportunities afforded to human foragers by the development of ecologically homologous plant niches. From ~28,000 to ~10,200 cal BP a prominent feature of the southern Argolid peninsula was the existence of large swathes of coastal plains that were well-watered by seasonal and permanent streams and karstic springs. Postglacial climate amelioration led to their progressive colonization by junipers, annual legumes and grasses, almonds and wild pears, alongside patches of terebinths and wild plums/cherries growing at more humid localities. Unlike the rugged topography and highly fragmented hinterlands of the southern Greek mainland, the coastal shelf zone comprised numerous well-drained large terraces and steppe-like undulating surfaces, which provided optimal environments for the development of savanna-like vegetation. This highly distinctive biome likely extended not only on the coastal plain proximate to FC but all around the present-day coastline of the southern Argolid peninsula (Fig 15).

The loss of a large portion of the coastal shelf to SLR between the LGM and the start of the Younger Dryas at ~12,900 cal BP was counterbalanced by the dramatic expansion of grassland vegetation caused by the abrupt increases in temperature and precipitation that marked the start of the Holocene at ~11,700 cal BP [21]. This climatic peak boosted the availability of dense, highly productive stands of annual grasses and legumes alongside nuts and fruits during the Mesolithic period, which is amply demonstrated by the extraordinary density and diversity of the FC Lower Mesolithic botanical assemblage. However, continuous (if slow-paced) SLR between ~11,700–9000 cal BP eventually led to the demise of these unique coastal palaeohabitats. Although isolated patches of grass and open woodland vegetation probably persisted along the coast, on the FC terrace and on the hills and inland alluvial plains, these could no longer sustain intensive, year-round human foraging for food and firewood. The deterioration of traditionally exploited plant resources was probably exacerbated further during the early 9^th^ millennium cal BP by the increasing salinization of the Kiladha bay area due to saltwater upstream movement and intrusion of the aquifer via the coastal karst systems. Thus, it is likely that the introduction at ~8700 cal BP of domesticated emmer wheat and 2-row hulled barley could have been motivated, at least in part, by these irreversible shifts in local plant ecologies and palaeohabitats that brought about a significant reduction in the availability of hitherto exploited plant resources. The integrated wood and non-wood botanical datasets point to a complete shift in the ecology of fuel wood gathering and food production at the very beginning of the 8^th^ millennium cal BP (possibly earlier, albeit no charcoal data are available from the Final Mesolithic and Initial Neolithic periods). As the FC coastline approached its modern configuration, fuel wood gathering targeted Mediterranean maquis woodlands that were also routinely cleared for the establishment of cereal crop fields. Cereal cultivation provided the bulk of plant-derived subsistence in the Neolithic period, with a concurrent significant reduction in the consumption of nuts and other fruit species gathered from the wild.

Whether or not the prehistoric inhabitants of FC cultivated wild cereals and legumes during the late Palaeolithic and the Mesolithic (e.g., as part of niche construction activities at times of plant resource abundance or, conversely, in response to cumulative resource stress caused by SLR) is a question that cannot be addressed at present without new excavation and primary archaeobotanical data collection. Despite the large scale and the intensity of sampling at FC in the 1970s, the large flotation mesh size used at the time has resulted, at best, in the sporadic recovery of certain elements of the small-seeded wild flora (e.g., *Medicago* seeds). This non-rectifiable recovery bias precludes a more precise evaluation of the floristic composition of the FC grassland vegetation and how it changed through time, including the ruderal floras associated with intensively gathered (and, potentially, managed) crop progenitor species such as barley, oats and lentils. A new program of carbon and nitrogen stable isotope analyses of crop progenitor and other large legume seeds could also help elucidating plant growth conditions including identifying the impacts of a range of possible plant management regimes (e.g., planting, tillage, watering, soil enrichment, etc). The hypothesis of cultivation without domestication at FC (and potentially other sites too) must therefore remain open to future empirical testing. However, it should also be noted here that the FC archaeobotanical record is compatible with the pattern of pre-agricultural plant exploitation known from early Holocene habitation sites in Southwest Asia in which the selective introduction of crop domesticates was preceded by long periods during which wild-type grasses and/or legumes were managed without evidence for local domestication events or even “predomestication” cultivation [21–22].

The meta-analysis of the previously published non-wood botanical dataset has re-affirmed the early introduction of a limited set of 2 domesticated cereal crops (emmer wheat, 2-row hulled barley) which took place soon after 9000 cal BP and was followed in later Neolithic phases by the introduction of einkorn wheat [14]. Unlike previous studies concluding that domesticated lentils formed an integral part of a crop “package” imported into FC from the Near East, the present study has uncovered tantalizing hints suggesting that large-seeded legumes (including lentils) were not incorporated in the FC Neolithic agroecologies as crops. In turn, the apparent absence of pulse cultivars from the FC Neolithic cultivation systems (or their low-level contribution to them) points to a complete break with Mesolithic traditions of plant management, in which lentils held a prominent position. It also presents a poor fit with a pattern of long familiarity with the exploitation of large-seeded legumes that can be traced as far back as the Lateglacial. The non-wood botanical record thus appears to be in full agreement with the results of faunal analyses indicating the wholesale replacement of Mesolithic broad-spectrum prey choice by a domestic animal economy focused on caprine meat production [19]. However, unresolved discrepancies remain between the (botanical and faunal) subsistence archaeology records and at least some material culture data categories (including chipped stone, marine mollusks and personal ornaments). The latter point to cultural continuities between the Final Mesolithic and the Initial Neolithic and have been interpreted as indicators of acculturation rather than outright colonization by Neolithic farming groups migrating from the Near East [20]. A more productive way to address these apparent contradictions in the FC archaeological record is through the hypothesis that domesticated cereal crops were selectively introduced at FC, alongside herded caprines, as part of a complex pattern of cultural interactions that brought together indigenous and immigrant groups. The non-wood botanical data are strongly suggestive of the idiosyncratic nature of FC Neolithic crop choice. The fact that (unlike other Neolithic sites in Greece) einkorn wheat was introduced to FC only after the Early Neolithic has already been noted in previous publications [14]. Seen in this light, the apparent exclusion of lentils from the core group of FC crop cultivars (even though lentils were already widely cultivated across Southwest Asia during this period) appears less extraordinary. In this context, it is no longer possible to speak of a single crop “package” that moved westwards as part of the toolkit of a group of pioneer settlers from the East. Instead, it may be more productive to explore the selection and use of specific cultivars as innovations that were negotiated between local and immigrant actors (e.g., individuals, kin groups or larger communities) and were incorporated to site- or area-specific social practices (e.g., through mate exchange networks). Such a hypothesis could be further explored through contextual analyses of the existing FC material culture record, alongside future fine-grained archaeobotanical sampling of closely controlled archaeological contexts.

A broadly comparable pattern of selective crop adoption has been recently proposed for interpreting the introduction of domesticated crop species at the site of Boncuklu in the Konya plain of central Anatolia during the 11^th^ millennium cal BP [36]. A key difference between Boncuklu and FC is that at the former crops were selectively incorporated as minor components in the local hunter-forager economy that (apart from nuts, hackberries and, possibly, some wetland plants too) exhibited no interest in the intensive exploitation of cereal crop progenitor taxa, despite their modelled wide distribution in the Konya plain during the early Holocene [37] also suggested by their very sporadic presence in the Boncuklu botanical assemblage [36]. By contrast, the introduction of domesticated cereal crops and herded caprines at FC in the early 9^th^ millennium cal BP took place against a background of SLR-induced cumulative habitat erosion that led to the decimation of the traditionally exploited plant and faunal resources. Domesticated cereals and caprines thus acted as catalysts for the reorientation of the local economies, in what was by then a low-yield and ecologically highly fragmented Mediterranean coastal landscape that could no longer sustain the exploitation of wild plants and game as staple foods by human foragers. The deep history of such processes of selective plant resource adoption and spread can be traced in the increasing circulation of population groups, resources and knowledge in the Eastern Mediterranean during the early Holocene. Such movements were already underway during the “long” 11^th^ millennium cal BP (exemplified by the transference of cultivars, animals and Anatolian obsidian into Cyprus, and of domesticated crop species into central Anatolia) and peaked with the intensification of maritime interaction networks (centered on Melian obsidian, cultivars and domesticated caprines) in the Aegean basin from the 9^th^ millennium cal BP [38] (see also Fig 1A).

## Materials and methods

Excavations at Franchthi Cave were conducted between 1969–1976 by T. W. Jacobsen of Indiana University and M. H. Jameson of Pennsylvania University under the auspices of the American School of Classical Studies in Athens in collaboration with the Greek Archaeological Service. The archaeobotanical samples generated by these excavations (including the wood charcoal specimens reported in this paper) were exported in 1982 for analysis outside Greece with the permission of the Hellenic Ministry of Culture (permit no. 39573/468). The wood charcoal specimens reported in this paper (a full list by excavation unit number is included in S1 Data File, and by location and excavation unit number below, under the heading “Sample selection, sub-sampling and charcoal identification”) were clean-sorted by Julie Hansen in the 1980s from the flotation samples she analyzed as part of her doctoral dissertation focusing on charred non-wood (seed and fruit/nut) remains. They represent approximately 1/4 of the wood charcoals originally sorted by Hansen [14: p.26] and are stored at the Archaeobotany Laboratory of the University of Liverpool (Liverpool, UK) and at the Boston University Environmental Archaeology Laboratory (Boston MA, US) where they can be accessed upon request.

### Field recovery methods

Most archaeobotanical samples from which charcoal macrofossils were available for analysis were retrieved in the field in the 1970s through machine-assisted tank flotation using the local spring water supply. Large-scale tank flotation was systematically applied from the 1971 excavation season, in principle targeting 100% of all excavated deposits in trenches F/A and H/H-1 [14, 39]. There was no systematic recording of the exact volume of soil processed from each excavation unit [14]. Both the flot and the heavy residue (HR = non-floating) fractions were captured in ~1.5mm plastic meshes. By modern standards (flot mesh size of 0.250mm, with HR mesh 0.5mm) this mesh size is considered too large to achieve consistent retrieval of all size classes of botanical remains including small-seeded grasses and legumes, other wild seeds and chaff. However, it has had a minimal impact on the retrieval of wood charcoals that are normally sub-sampled in the lab from charcoal fragments >4->2mm [23]. A minority of archaeobotanical samples had been processed by Jane Renfrew in the 1969 excavation season using small-scale bucket flotation. These comprised small (unspecified) volumes of sediment retrieved from select excavation units, which were first passed through a stack of dry “shaker” sieves (mesh sizes 12, 6 and 3.5mm) and then poured into water and the floating charred plant remains scooped out with a small hand sieve or poured into a fine cloth mesh [14, 39].

### Sample selection, sub-sampling and charcoal identification

The anthracological specimens available for analysis originated in trenches H1A (excavated to a depth of -9.26m) and FAS (excavation stopped at a depth of -11.2m due to reaching sea level) (see also Figs 3–5). A batch of 18 charcoal samples (NB: throughout this paper the term “charcoal sample” refers to the wood charcoal component of the archaeobotanical flotation samples originally sorted by Julie Hansen) from trench H1A (excavation units 161–162, 164–179) and 2 charcoal samples from FAS (173–174) had been transferred by Hansen in the 1990s to the Institute of Archaeology, University College London for the analysis of charred parenchyma tissues by Jon Hather. These charcoal samples were retrieved from the UCL stores in 2006 and were subsequently studied by Asouti at the Archaeobotany Laboratory of the Department of Archaeology, Classics and Egyptology, University of Liverpool. A second batch of charcoal samples from H1A (165, 170, 173, 179–181, 183, 187–194, 196, 199–200, 205–210, 212–213, 215, 218–219; including some split samples equivalent to the samples located in the UK), the H1A samples that had been retrieved in 1969 using small-scale bucket flotation (100–101, 106, 109, 132–133, 135, 137–140, 142–155, 157, 160), the FAS 175 and split samples 173–174, and the FAS samples (73, 75–76, 82, 87, 89, 98, 101, 112–113, 117–118, 121, 124, 126) representing part of the FC Neolithic sequence were deposited at the Department of Anthropology of Boston University. These charcoal samples were studied by Ntinou under the auspices of the M.H. Wiener Laboratory of the American School of Classical Studies at Athens.

Microscopy work focused on all wood charcoal fragments >4mm to minimize potential identification biases (i.e., over-representation of certain taxa such as oak that are easy to identify even when present with very small fragments, or of very small indeterminate fragments). Taxon diversity saturation curves were obtained for every wood charcoal fraction containing more than 100 fragments >4mm with counting stopped once curves had levelled off. An exception was made for the >4mm charcoal fractions of samples FAS:173–175. These were the only excavation units available for analysis from stratum W2 (Lower Mesolithic) that had been sampled with large-scale tank flotation. Hence, their charcoal counts were raised as much as feasible in order to enhance the representativeness of taxon diversity for this segment of the charcoal stratigraphy.

**Fig 16:**
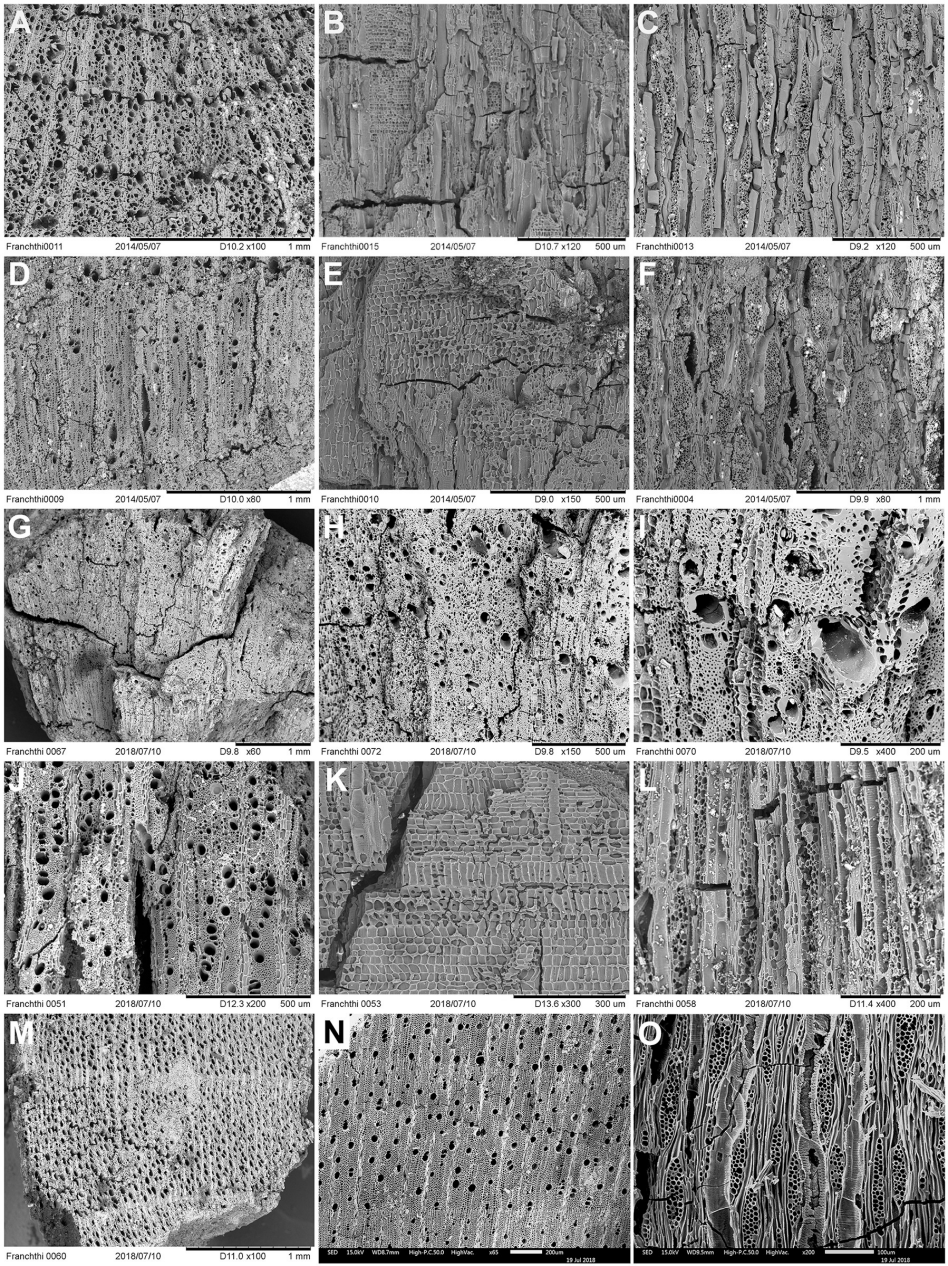
SEM images of wood charcoal taxa from Franchthi Cave. (A-C): [H1A:164] *Amygdalus* 3-4-5seriate–TS, RLS, TLS planes; (D-F): [H1A:164] *Amygdalus* small round wood/twig with sparse pores and very wide rays 5 to >10seriate–TS, RLS, TLS planes; (G-F): [H1A:179] *Amygdalus* with very sparse pores–TS planes; (J-L): [H1A:165] *Prunus* (diffuse porous) with 1-2/3-4seriate rays–TS, RLS, TLS planes; (M): [H1A:170] Maloideae–TS plane showing distinctive terminal narrow bands of flattened cells in the latewood, an indicator of severe seasonal moisture deficit impacting growth pattern during the dry season; (N-O): [FAS:118] *Acer*–TS, TLS planes.

**Fig 17:**
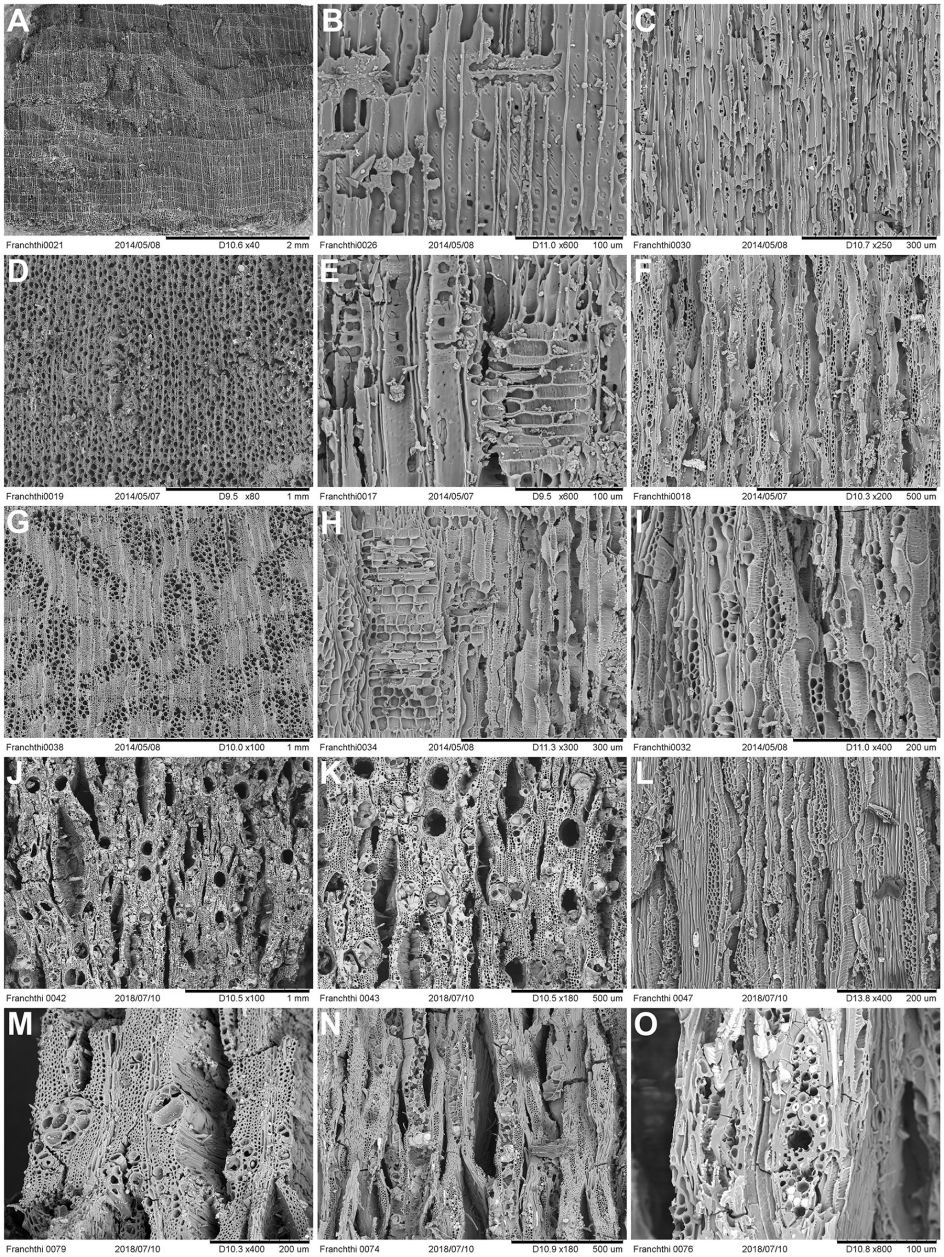
SEM images of wood charcoal taxa from Franchthi Cave. (A-C): [H1A:172] *Juniperus*–TS, RLS, TLS planes; (D-F): [H1A:164] Maloideae–TS, RLS, TLS planes; (G-I): [H1A:164] *Rhamnus/Phillyrea*–TS, RLS, TLS planes; (J-K): [H1A:167] *Pistacia*–TS plane; (L): [H1A:162] *Pistacia–*TLS plane; (M-O): [H1A:166] *Pistacia*–TS, TLS planes.

**Fig 18:**
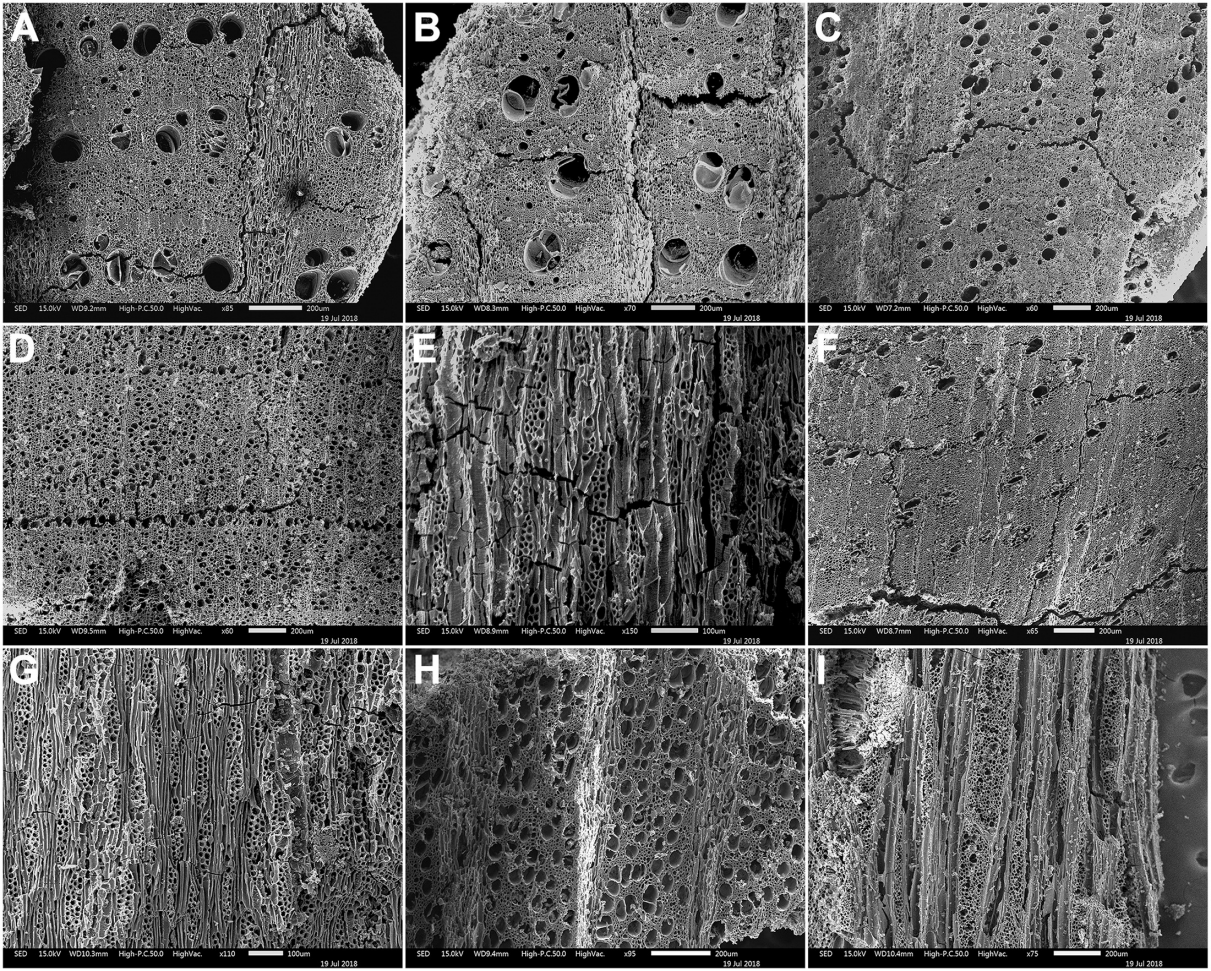
SEM images of wood charcoal taxa from Franchthi Cave. (A): [FAS:121] *Quercus* (deciduous)–TS plane; (B): [FAS:126] *Quercus* (deciduous)–TS plane; (C): [FAS:112] *Quercus* (evergreen)–TS plane; (D): [FAS:112] *Arbutus*–TS plane; (E): [FAS:118] *Arbutus*–TLS plane; (F-G): [FAS:112] *Fraxinus*–TS, TLS planes; (H-I): [FAS:112] *Platanus*–TS, TLS planes.

Depending on their size, wood charcoal specimens were either hand- or pressure-fractured with a carbon steel razor blade to produce fresh sections of all three anatomical planes (TS = Transverse Section, RLS = Radial Longitudinal Section and TLS = Tangential Longitudinal Section). These were examined using incident-light (darkfield/brightfield) materials optical microscopy at magnifications x50, xl00, x200, x400-x500. Botanical identifications (to genus or family level) were made using the wood anatomical identification criteria established for European and Eastern Mediterranean arboreal floras. Select charcoal fragments were also compared to specimens held in modern wood charcoal reference collections in Greece and at Liverpool. Part of microscopy analysis also involved the evaluation of some of the charcoal identifications previously published by Hansen. Wood charcoals previously reported as *Quercus pubescens*-type [14] were found to represent *Amygdalus* instead. This fact (alongside the results of the much more comprehensive anthracological analysis presented in this paper) positively disproves previously published claims [14] for the prevalence of an open oak woodland at FC during the late Upper Palaeolithic and the Mesolithic. SEM microphotographs of select charcoal taxa found at FC are presented in Figs 16–18.

All charcoal fragments identified as *Juniperus* by the present study exhibited ray height (TLS) predominantly in the range of 1–4 cells. Based on the ubiquity of this anatomical feature we classified them as *Juniperus* sp. instead of *Cupressus* (cypress) which belongs to the same family (Cupressaceae) and in charred form exhibits almost identical wood anatomical characters to *Juniperus*. All *Pistacia* wood charcoals exhibited ray width (TLS) predominantly in the range of (2)3-4-5(6) cells. We found no specimens with predominantly narrow rays (1-2-3seriate) that would have indicated the presence of *Pistacia lentiscus*, the species originally proposed by Hansen based on her observations of the surface morphology of charred *Pistacia* nutlets [14]. The morphology of *Pistacia* nutlets is highly variable. Although in principle both *P*. *terebinthus* and *P*. *lentiscus* could be represented at FC, it is improbable that fuel wood was procured exclusively from *P*. *terebinthus* and nuts from *P*. *lentiscus*. For this reason, it is more likely that the species represented in the wood and non-wood charred plant assemblage is *Pistacia terebinthus*.

The distinction drawn between *Amygdalus* and *Prunus* has its basis on modern wood anatomical studies that separate between two broad taxonomic groups: (1) *Amygdalus webbii*, *A*. *dulcis* (ring-porous = RP, with ray width 3–5 or 6–8[10]seriate) and (2) *Prunus avium*, *P*. *cerasus*, *P*. *spinosa*, etc. (diffuse to semi-ring porous = DP, with pores arranged mostly in radial files and predominantly narrow rays 1–3 or 3-5seriate) [40]. The same wood anatomical distinction is replicated in the modern charred wood reference specimens held at Liverpool, including the aforementioned species plus *A*. *orientalis*, *A*. *korshinskyi*, *P*. *cerasifera* and *P*. *microcarpa*, and has also been verified in other Eastern Mediterranean late Pleistocene and early Holocene charcoal assemblages in which RP only, or RP and DP morphotypes have been detected [9, 29, 41–42]. Separating *Amygdalus* from *Prunus* based on wood anatomy also agrees with previous and more recent botanical and genetic work in the Eastern Mediterranean and Southwest Asia that treats *Amygdalus* as a distinct genus from *Prunus* within the Rosaceae family [43]. In the FC charcoals we identified 2 different growth-ring morphotypes (ring-porous = RP, corresponding to *Amygdalus* and diffuse-porous = DP, corresponding to *Prunus*). We thus concluded that both *Amygdalus* (RP) and *Prunus* (DP) were present in the charcoal samples. All intermediate (semi-ring porous) types that could not be characterized more precisely due to the small size of the analyzed specimens were classified as Prunoideae (used to denote *Prunus/Amygdalus* indet). Further separation between individual species of *Amygdalus* is not possible based on wood anatomy. Hansen’s analysis of *Amygdalus* charred nutshell remains suggested that *Amygdalus webbii* is more likely to be represented rather than *A*. *communis* [14]. Wild *A*. *webbii* (syn. *Prunus webbii* (Spach) Vierh.) is native to the southern Balkan peninsula, including mainland Greece, the eastern Aegean islands, and the island of Crete. It is thus likely (if unprovable) that *A*. *webbii* is present in the FC charred plant assemblage both as charred wood and nutshell fragments.

### Recording of dendroanthracological features

**Fig 19:**
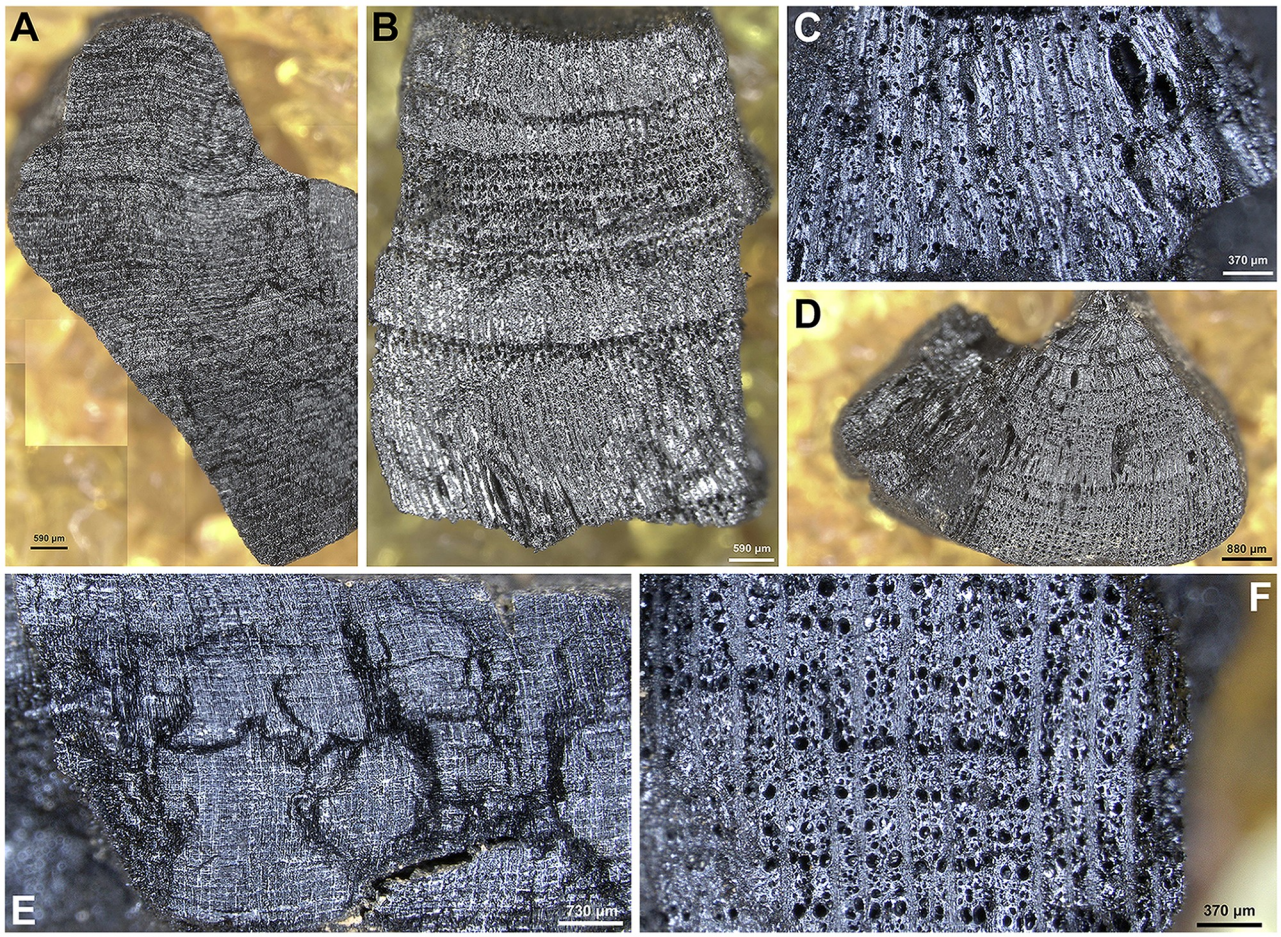
Stereozoom stacked and stitched digital microphotographs of wood charcoal taxa (TS planes only). (A): [H1A:166] *Juniperus* old stem wood with extremely narrow growth rings indicating very slow growth under dry and cold conditions [45]; (B): [H1A:171] *Amygdalus* round wood showing successive years of suppressed growth (narrow rings) after cutting, followed by period of growth release [45]; (C): [H1A:176] stacked and stitched image of *Amygdalus* round wood showing large rays and sparse pore distribution; (D): [H1A:167] *Amygdalus* large twig/small round wood: similar to (B) with bands of narrow rings indicating that it originated from a plant that was repeatedly cut [45]; (E): [H1A:175] similar to (A); (F): [H1A:175] *Amygdalus* stem wood with very narrow growth rings indicating slow growth under cold and arid conditions.

Several wood anatomical characters and eco-anatomical features were recorded for each botanically identified fragment >4mm including curvature degree (including 3 classes: CD1 = large caliber charcoal originating from stem or large round wood, CD2 = intermediate caliber, CD3 = small caliber charcoal originating from twigs or small round wood) and the presence/absence of pith and bark, resin/gum ducts, fungal hyphae, scar/callus tissue, and narrow/false rings. The aim was to explore woodland growth conditions (including evidence of growth as shrubs or trees, and environmental stress) and potential preferences for the gathering of small-size and dead/decayed wood as fuel [44]. Kabukcu designed the protocol for the recording of the FC dendroanthracological features. Ray width was recorded for most taxa, but was systematically quantified only for *Amygdalus*, to explore if the occurrence of specific ray width classes could be related to genetic differences or is associated instead with wood caliber. Vitrification was ubiquitous in *Amygdalus* charcoals hence, although this feature was recorded, it was not quantified. TS planes of select specimens from the dominant taxa (*Amygdalus*, *Juniperus*, Maloideae) were photographed using a Leica S8APO stereozoom microscope fitted with a GXCAM HICHROME-LITE HDMI 5MP camera at the Liverpool Archaeobotany Laboratory to create a set of reference images of the eco-anatomical features observed on the TS planes of the major charcoal taxa present at FC (Fig 19). A systematic comparative study of *Amygdalus* eco-anatomy from FC and other late Pleistocene and early Holocene sites in the Eastern Mediterranean (including dendroanthracological features plus continuous growth ring width and pore size/density measurements) is currently underway and will be published elsewhere.

### Data reduction and stratigraphic analysis methods

The primary (sample-by-sample) charcoal taxon counts are presented in spreadsheet form in S1A Data File. Total numbers of examined fragments and identified fragments are given for each excavation unit, plus total numbers of identified fragments for every FC stratum represented in the anthracological sequence. Grouping samples by stratum was the principle means for linking the charcoal stratigraphy to the FC archaeological chronology (see Figs 4–6 and S1A and S1B Data File). This is because the FC lithostratigraphy provides a robust stratigraphic framework that is largely replicated across trenches and is moreover radiocarbon-dated. The correspondence of the sampled excavation units to the FC general phases (FGPs) and (for the Neolithic period) cultural phases (CPs) is also denoted, to facilitate comparisons with other categories of archaeological materials.

To date, there has been no detailed published description of the archaeological features excavated at FC. For this reason, all sampled excavation units were considered, in principle, to represent generalized charcoal scatters found in variable concentrations across the sampled sequence, except for a few units that were unequivocally identified as hearths by the excavators (S1A Data File). However, the highly variable >4mm charcoal counts recorded for several H1A sampled units also indicated the probable existence of differences in context-and/or layer-specific taphonomic pathways that remain unaccounted for in the published descriptions of the FC stratigraphy. The inspection of the published sections, especially the H1A and FAS schematic sections [13: Plates 32–33, 35–36] that represent in approximation the relationships between the different excavation units within each trench, revealed that several H1A units were contiguous (hence denoting artificially split layers and/or distinct contexts/features dug within the same layer) or cross-cutting (thus pointing to distinct depositional events accommodated within the same archaeological horizon/layer). Thus, to minimize the risk of creating false patterns by artificially separating into different units the same stratigraphic event, we proceeded with amalgamating the charcoal counts derived from all such units. The purpose was to restore the stratigraphic integrity of the charcoal sequence, which in turn was a prerequisite for producing reliable reconstructions of diachronic trends in charcoal sample composition and interpreting them as palaeoecological proxies. To this end, all 92 sampled excavation units were allocated to 35 Charcoal Stratigraphy Units (CSUs). Excavation units that were represented in the published schematic sections as split or cross-cutting layers and/or superimposed very fine lenses were amalgamated in respective CSUs as appropriate. Equally, every excavation unit that was dug as a well-defined uninterrupted layer (“pass”) or an archaeological feature was transposed to a single CSU. In some instances, it was necessary to amalgamate blocks of otherwise well-defined excavation units into single CSUs. This was the case with strata R and S1, due to the extremely low charcoal counts retrieved from their constituent units. Amalgamating their charcoal counts was the only way to capture effectively charcoal taxon frequencies for the timespans represented by R and S1, while also respecting the stratigraphic integrity of this segment of the charcoal stratigraphy. The same, albeit slightly modified, principle was applied to the H1A excavation units originating in strata W1 and W2, which also had comparatively low charcoal counts (all had been sampled for plant remains pre-1971 using small-scale bucket flotation). All H1A stratum W1 units were amalgamated in a single CSU. H1A stratum W2 units were sub-divided in 2 CSUs by drawing an arbitrary distinction between the lower and upper parts of W2 as described in the published schematic sections. For H1A stratum X2 and FAS strata Y2-3 the stratigraphic position of the sampled excavation units was either uncertain (X2) or the charcoal sample coverage of the archaeological sequence was discontinuous (Y2-3). In these cases, we used the cultural phases (CPs) to amalgamate individual units into chronologically coherent CSUs. This was particularly useful for the Neolithic strata Y2-3 sampled in trench FAS, as it permitted distinguishing between middle, late and final Neolithic CSUs. The full details of the allocation of all sampled FC excavation units into CSUs are presented in S2 Data File.

### Construction and phasing of the anthracological diagram

CSUs provided the baseline data (units of analysis) with which to construct the FC anthracological diagram. Further data reduction was necessary involving the exclusion of indeterminate (Indet.) fragments from the per-CSU charcoal counts. In all cases, these were charcoal fragments that were unidentifiable due to their very small size and/or poor preservation. They could only be determined as Indet. conifers (Indet. C) or angiosperms (Indet. A). Including them in the quantified charcoal counts would have provided no useful information for palaeoenvironmental reconstruction (also considering the very low Indet. C fragment count (= 5) recorded for the entire anthracological sequence). The Indet. category does not include unidentifiable taxa (i.e., charcoal fragments preserving a sufficient range of anatomical characters, which were not botanically identified to genus or family due to the lack of adequate reference material; no such fragments were present in the FC charcoal assemblage). The occasional bark fragments were also excluded from the per-CSU charcoal counts, alongside fragments of *Quercus* spp. which (due to their small size) were impossible to determine as either deciduous (D) or evergreen (E) oak. These were mostly present in Neolithic (strata Y2-3) samples, in which both evergreen and deciduous oak charcoals are well represented. Thus, including them in the anthracological diagram could not add something significant to palaeoenvironmental reconstruction. Another *Quercus* spp. fragment recovered from H1A:207 is almost certainly intrusive from stratigraphically later units hence it was also excluded. By contrast, other highly likely intrusive fragments (1 fragment of *Arbutus* in H1A:213 and 1 fragment of *Quercus* (E) in each of H1A:207 and H1A:160) were kept in since the same taxa also appear in Upper Mesolithic (evergreen oak) and Neolithic (both taxa) CSUs. Their status as intrusive is clearly marked in the data tables (S1A and S3 Data Files) and in the anthracological diagram (Fig 6).

The anthracological diagram (Fig 6) was generated by Kabukcu using the R statistical software (version 3.5.0, package ‘*rioja*’, function ‘strat.plot’) and edited by Asouti (with the addition of labels and phasing boundaries) with Adobe Photoshop CC 2018. It represents the FC anthracological sequence from the earliest (H1A:219–212 –Stratum R) to the latest CSU (FAS N.V.2 –Stratum Y3) and covers a timespan of ~22,000 years including all FC strata, except for W3 and X1 (Final Mesolithic) and X2 (Initial Neolithic; except for trench H1A in which X2 comprises Upper Mesolithic deposits) from which no charcoal samples were available for analysis (S1B1-2 Data File). 5 Charcoal Phases (C-Phases 1–5) were identified, based on observed changes in charcoal assemblage composition. Sub-phases 2a-b, and 4a-b were distinguished based on the same principle. By contrast, separating sub-phases 4c-e was dictated by the fact that (although stratigraphically contiguous with the rest of the H1A sequence) samples from excavation units H1A:160–144 (4c) were affected by inherent recovery biases (sub-optimal bucket flotation sampling). Thus, the taxon frequencies derived from H1A:160–144 were considered as un-representative of the relative proportions of the charcoal taxa originally deposited in these units. The same principle applied to the excavation units included in CSU H1A (VIII) that was also treated as a separate sub-phase (4e). Units FAS:175–173 (although belonging to stratum W2) derived from a different trench. They were thus grouped in sub-phase 4d in recognition of the fact that they represent a floating segment of a different (FAS) stratigraphic sequence, that in theory could have been affected by different (if unknown) taphonomic factors when compared to the H1A Mesolithic sequence. Lastly, no attempt was made to distinguish sub-phases within C-Phase 5 (CSUs FAS N.II, IV, V) as the charcoal sample coverage for this part of the FAS stratigraphic sequence was discontinuous.

### Multivariate analysis methods

Kabukcu designed and performed all multivariate statistical analyses. To gain a better understanding of vegetation change through time, it was necessary to integrate the results of the anthracological analysis with those of the analysis of non-wood botanical remains previously published by Hansen [14]. To this effect, a Correspondence Analysis (CA) was performed on the per-sample charred wood and non-wood taxon counts. The underlying principle is that botanical remains recovered from the same samples are more likely to have derived from the same vegetation catchment; i.e., that fuelwood gathering took place in broadly the same localities and/or habitats as plant-food foraging during the different phases of prehistoric occupation [41]. CA has been previously applied to integrated archaeobotanical datasets [41–42, 44] with the aim of detecting patterning in large and complex sample populations. As one of the most widely used ordination techniques, CA allows for a 2-dimensional evaluation of complex patterning in a dataset by means of geometric representation along two axes. In the present study, CA was applied on the charcoal and non-wood taxon count matrix using R (version 3.5.0, package ‘*FactoMineR*’).

To minimize the impact of taphonomic biases on the multivariate data matrix a series of data reduction actions were performed on the FC non-wood botanical dataset. All taxa that were demonstrably preferentially preserved (e.g., high counts of mineralized Boraginaceae seeds) were excluded from the data matrix. Only whole item (or whole equivalent) counts were used. Counts of chaff, pod fragments, and unconverted seed or other plant part fragment counts were also excluded from the data matrix, in order to minimize the risk of taxon over-representation through these particular data categories. Likewise, for *Amygdalus* and *Pistacia* nutshell Hansen’s original MNI (Minimum Number of Individuals) converted counts were kept in, while any unconverted fragment counts were rejected. For the wood charcoal counts, only positively identified fragments (to genus or family level) were included (hence excluding all “cf.” identifications from the data matrix). Data reduction at the sample level involved limiting analysis only to samples that contained >20 identified items (where 20 equals the sum of the non-wood and wood identified items) to reduce redundancy and noise in the resulting data matrix.

The dendroanthracological dataset was analyzed with Multiple Correspondence Analysis (MCA) using R package ‘*FactoMineR*’ [44]. MCA is an ordination technique similar to CA, which is suited to the analysis of multiple categorical variables, and results in a similar reduction of dimensions in a complex dataset. The analysis was carried out using individual specimens as data points (each having been assigned a unique identifier: Trench/Sample number/fragment number) to investigate the co-occurrence of individual features and taxa and explore any associations between the different qualitative variables (dendroanthracological features) recorded.

Given the comparatively low number of samples from which both wood and non-wood botanical data were available, and the inherent recovery biases affecting the greater part of the Lower and Upper Mesolithic botanical sequence from H1A and FAS, it was necessary to expand multivariate analyses to include archaeobotanical samples from which no charcoal samples were available for analysis, thus incorporating materials originating from trenches FAN and H1B. The aim was to enhance as much as possible our understanding of changes in non-arboreal flora and vegetation through time, and the types of plant palaeohabitats exploited by the FC inhabitants through its long occupation history. The same data reduction methodologies outlined above were repeated for this sample population. In the resulting data matrix, all taxa and samples counting <10 identified items were excluded, in order to reduce the degree of redundancy and prevent patterning forced by extremely rare taxa and/or low-density samples. A pervasive feature of the FC archaeobotanical assemblage is the enormous variation observed in botanical finds density (which is due, at least in part, to taphonomic factors) across the different occupation phases. This characterized especially the Lower Mesolithic samples (strata W1-2) that contained >28,000 identified items, contrasting with the considerably lower botanical densities retrieved from both later and earlier samples [14]. For this reason, Nonmetric Multi-Dimensional Scaling (NMDS) was used to evaluate the non-wood botanical data matrix. NMDS is a method widely applied in ecological analyses of taxon abundance counts, particularly when dealing with inconsistencies in taxon preservation and/or sampling [46]. Its application at FC allowed us to summarize more effectively large-scale patterning in sample composition and to examine associations between wild taxa through time. The data matrix (per-sample taxon counts) was transformed with the Bray-Curtis index. Using the R package ‘*vegan*’, function ‘metaMDS’, a 3D plot of the individual samples and projected species values was produced [46]. The results and efficiency of scaling were evaluated by examination of the Shepard stress plot (see Fig 11) and the reported stress-value (= 0.165) (following Clarke 1993 [46] stress values are classified as <0.05 = excellent, <0.10 = good, <0.20 = usable, >0.20 = not acceptable).

## Supporting information

S1 Data File (A) Absolute charcoal fragment counts for all excavation units sampled from FC Trenches H1A and FAS; (B-1) Botanical zones as defined by Hansen [14] and their correlation with sampled FC excavation units; (B-2) Correlation of FC excavation units that have produced charred plant remains with lithostratigraphic units (FC Strata).
(XLSX)

S2 Data File Definition of the Charcoal Stratigraphy Units (CSUs) used for constructing the FC anthracological diagram. (XLSX)

S3 Data File Baseline data for the FC anthracological diagram: Absolute and percentage charcoal fragment counts of FC CSUs grouped by Charcoal Phase (C-Phase). (XLSX)

S4 Data File (A) Dendroanthracological data for 1344 charcoal fragments from trenches H1A and FAS; (B-1) Results of Multiple Correspondence Analysis (MCA) run on dendroanthracological features on all fragments: eigenvalues, percentage of variance and cumulative percentage of variance for each dimension; (B-2) Results of Multiple Correspondence Analysis (MCA) run on dendroanthracological features: contribution of variables to dimensions, and cos2 values.
(XLSX)

S5 Data File (A) Dendroanthracological data (CD, Bark, Pith and Ray Width) for 502 *Amygdalus* charcoal fragments; (B-1) Results of Multiple Correspondence Analysis (MCA) run on *Amygdalus* dendroanthracological features: eigenvalues, percentage and cumulative percentage of variance for each dimension; (B-2) Results of MCA run on *Amygdalus* dendroanthracological features: contribution of variables to dimensions, and cos2 values.
(XLSX)

S6 Data File (A) Data matrix (taxon counts) for FC samples containing both wood charcoal and non-wood charred plant remains (trenches H1A, FAS); (B-1) Results of Correspondence Analysis (CA) run on the per-sample charcoal and non-wood botanical taxon counts: contribution to dimensions and cos2 values; (B-2) Results of CA run on the per-sample charcoal and non-wood botanical taxon counts: eigenvalues and percentage of variance for dimensions.
(XLSX)

S7 Data File Data matrix (taxon counts) for all FC samples containing non-wood charred plant remains (all trenches).
(XLSX)
